# Exceptional point-enhanced piezoelectric thermometry via anti-parity−time symmetry

**DOI:** 10.1038/s41378-026-01353-7

**Published:** 2026-06-15

**Authors:** Jiajun Wang, Jie Li, Bei Wu, Xiwei Huang, Wenjun Li, Jikui Luo, Minye Yang, Shurong Dong, Weipeng Xuan

**Affiliations:** 1 College of Electronics and Information, Ministry of Education Key Laboratory of RF Circuits and System, Hangzhou Dianzi University, Hangzhou, China; 2 College of Information Science and Electronic Engineering, Zhejiang University, Hangzhou, 310027 China; 3 International Joint Innovation Center, Zhejiang University, Haining, 314400 China; 4 School of Artificial Intelligence, Optics and Electronics, Northwestern Polytechnical University, Xi’an, 710072 Shaanxi China

**Keywords:** Electrical and electronic engineering, Materials science

## Abstract

Temperature monitoring underpins critical processes in electronics, energy systems, and biomedical applications. Piezoelectric resonant temperature sensors based on lead zirconate titanate (PZT) are widely favored for their rapid thermal response and compact size, yet their linear temperature dependence severely limits the sensitivity. Here, we introduce a novel approach to piezoelectric thermometry by applying non-Hermitian physics and anti-parity−time (APT) symmetry to PZT resonators. The system couples two PZT resonators through energy interactions with gain/loss, forming an APT-symmetric architecture that operates at an exceptional point (EP). This configuration dramatically amplifies minute temperature-induced perturbations, achieving an ultrahigh temperature coefficient of frequency (TCF) of −1500 ppm·K^-1^ indicating a 17-fold enhancement over conventional single PZT sensors (~88 ppm·K^-1^), and over twice the sensitivity of state-of-the-art resonant temperature sensors without sacrificing power consumption or device size. Experimental results confirm a 2× improvement in signal-to-noise ratio (SNR) and dynamic adaptability. Unlike its PT-symmetric counterparts, APT symmetry tolerates intrinsic mismatches between PZTs, enabling robust EP operation and reconfigurability across a wide temperature range. By uniting non-Hermitian physics with piezoelectric platforms, this work establishes a new framework for piezoelectric thermometry in advanced electronics, energy storage, and biomedical systems.

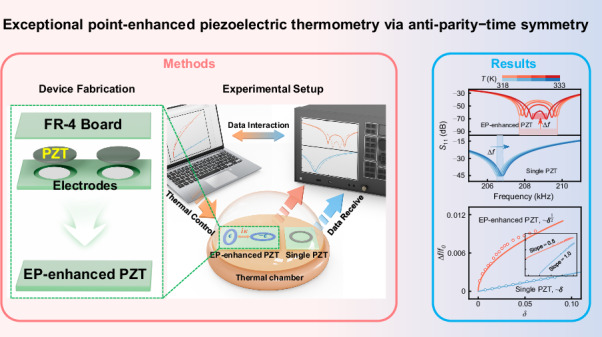

## Introduction

**Fig. 1:**
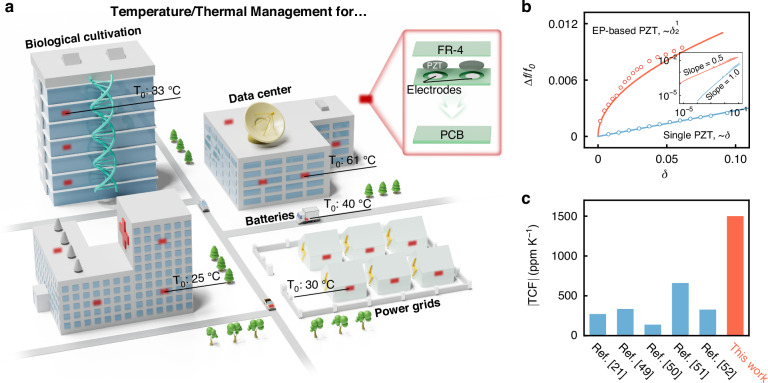
EP-enhanced PZT thermometry. **a** Diverse application scenario where the EP-enhanced PZT thermometry can be implemented. **b** Normalized eigenfrequency splitting $\Delta f$ of the EP-enhanced PZT sensor compared with a conventional single PZT sensor under the same temperature perturbation $\delta .$ Lines and circles here represent the theoretical and experimental data, respectively. In such a small perturbation limit, the EP-enhanced sensor outperforms the conventional one. **c** A summary of the absolute values of TCF produced by our system in comparison to state-of-the-art resonant temperature sensors

Temperature is a fundamental state variable across science and engineering, governing physical, chemical, and biological processes over a broad range of length and time scales. In biomedical systems, it informs diagnosis^1^, therapy^2^, and infection control^3^; in manufacturing, it stabilizes reaction kinetics^4^ and yield^5^; in energy systems, it governs efficiency^6^, ageing^7^, and safety^8^, spanning applications from batteries in electric vehicles to power grids; and in electronics, it sets performance limits, reliability, and thermal budgets, from server chassis in data centers^9^ to wearables^10^ (Fig. 1a). An accurate, spatially resolved, and time-responsive temperature readout is therefore of paramount importance, from cells and catalysts to battery packs and integrated circuits (ICs), making temperature sensing foundational to both fundamental studies and real-world applications.

State-of-the-art temperature sensing technologies have achieved widespread industrial adoption, notably exemplified by platinum resistance temperature detectors (RTDs), thermistors, thermocouples, and silicon-based ICs/micro-electro-mechanical systems (MEMS). These platforms have been widely implemented in process control, biomedical instrumentation, and embedded electronics by offering complementary advantages, including traceable accuracy and linearity (RTDs)^11^, high responsivity at low cost (thermistors)^12^, wide dynamic range (thermocouples)^13^, and compact, digitally calibrated readouts (ICs/MEMS)^14,15^. In parallel, emerging approaches, such as surface- and bulk-acoustic-wave (SAW/BAW) sensors^16^, chip-scale photonic/interferometric thermometry^17^, fiber Bragg gratings^18^, and pyroelectric/calorimetric microsystems^19^, have been explored to further improve sensitivity, bandwidth, and spatial resolution. However, despite these advances, their practical implementations remain constrained by calibration overheads, long-term drift, and the difficulty of simultaneously achieving high sensitivity, compact size, and low power consumption. Consequently, there is a growing demand for sensors that can tackle these challenges at once.

Piezoelectric resonant sensors, particularly those based on lead zirconate titanate $(\mathrm{PbZ}{\mathrm{r}}_{x}\mathrm{T}{\mathrm{i}}_{1-x}{{\rm{O}}}_{3},{\rm{PZT}}$), offer a promising route to address these challenges. Their appeal lies in supporting both resonant and impedance-based readouts within small footprints while offering rapid thermal response in thin-film and micro-resonator formats^20–22^. In resonant operation, temperature perturbs elastic constants, density, and permittivity, shifting the resonance frequency and phase^21^. Impedance-based sensing, in contrast, relies on temperature-induced variations in capacitance and dielectric loss, measurable via impedance analyzers^22^. These mechanisms enable PZT to combine miniaturization with energy efficiency, which is not possible in many alternatives. However, conventional PZT thermometry exhibits an essentially linear response over typical operating ranges (i.e., mode frequency or amplitude responds linearly to temperature), yielding limited sensitivity because of modest material properties. This linear-slope constraint imposes a significant trade-off between sensing resolution and speed: achieving sub-0.1 K resolution typically requires extended averaging or heavy data processing, both vulnerable to flicker noise and drift.

A compelling solution to this sensitivity limitation may arise from a novel framework rooted in quantum mechanics, namely, the non-Hermitian physics. It has been reported that systems operating near non-Hermitian exceptional points (EPs) can exhibit nonlinear responses to small perturbations $(\delta ):$ eigenfrequency splitting (∆*f*) obeys *N*^*th*^-root scaling ($\Delta f\propto {\delta }^{1/N}$ with *N* the EP order)^23–25^, amplifying minute parameter shifts without added power or footprint. These properties have enabled landmark applications, including single-particle detection^26–28^, optical gyroscopes^29–31^, and biomedical sensing^32–34^, to name just a few. While EPs can dramatically enhance sensitivity through nonlinear eigenfrequency responses, their practical implementation depends heavily on maintaining the underlying symmetry of the system, such as parity−time (PT) symmetry^25,31,35^ and chiral (sublattice) symmetry^36–38^. The classical PT-symmetric architectures rely on perfectly matched sub-oscillators. However, when utilizing distinct PZT elements, inevitable variations in fabrication and material properties inherently break this symmetry, thereby degrading performance^39,40^. In contrast, anti-PT (APT)^41–48^ symmetry provides a robust alternative: it tolerates intrinsic mismatches between two PZTs while preserving the EP characteristics.

Here, we implement a sensing architecture based on APT symmetry by coupling two distinct PZT resonators. The paired PZTs are engineered to exhibit nearly an identical loss rate and are linked through a resistor, satisfying the APT symmetry condition. By exploiting system eigenmodes rather than individual device parameters, minute temperature-induced perturbations in PZT material properties are nonlinearly amplified (Fig. 1b), significantly enhancing the sensitivity without increasing power consumption or footprint. As a result, the proposed APT-EP-enhanced PZT temperature sensor achieves an ultrahigh temperature coefficient of frequency (TCF) up to −1500 ppm·K^−1^$,$ which represents a 17-fold enhancement over the conventional single PZT sensor (88 ppm·K^−1^) and more than twice the sensitivity of state-of-the-art resonant temperature sensors^21,49–52^ (Fig. 1c, typically weaker than −700 ppm·K^−1^) while maintaining compactness. The system attains a limit of detection (LOD) down to 0.03 K and exhibits a marked improvement in signal-to-noise ratio (SNR) compared to conventional single PZT controls. Unlike PT-symmetric designs^33,34^, our approach does not require perfectly matched PZTs because of the APT symmetry, which allows frequency detuning between two PZTs while acquiring purely real eigenfrequencies beyond the corresponding EPs. Based on the temporal coupled-mode theory (CMT)^53,54^ analysis and the experimental validations in Supplementary Note VII, we demonstrate that this characteristic accommodates intrinsic fabrication discrepancies in PZTs, significantly relaxing degeneracy requirements for constructing EP-enhanced PZT sensors. Furthermore, this architecture offers high reconfigurability, allowing adaptation to diverse temperature ranges (from 293 K to 353 K) across various scenarios thanks to the APT symmetry. Utilizing non-Hermitian physics and PZT elements, this work presents a robust pathway for EP-enhanced piezoelectric thermometry, overcoming the inherent disadvantage of sensitivity, and enabling its applications in advanced electronics and energy systems.

## Results

In this section, we first establish the APT-symmetric architecture using two coupled PZT resonators and demonstrate the existence of an EP in the proposed electrical circuit. We then elucidate the EP-induced nonlinear response to temperature perturbations and experimentally benchmark the sensing performance against a conventional single-PZT resonant thermometer. Finally, we evaluate the SNR, LOD, and real-time temperature monitoring capability of the APT-EP enhanced PZT temperature sensor under representative practical scenarios.

### APT-symmetric EP using two PZTs

**Fig. 2:**
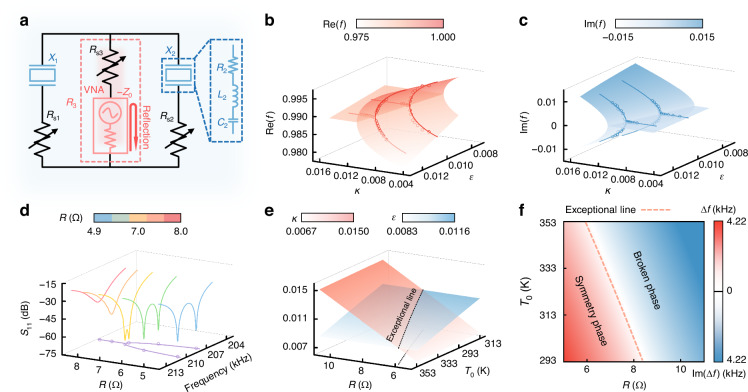
EP-enhanced PZT sensor and its characterization. **a** Circuit schematic of the APT-symmetric system composed of two PZT resonators, each modeled by the BVD equivalent network, with parameters included in Methods and Supplementary notes. Two PZTs are coupled through an effective resistive interaction $({R}_{3})$ while maintaining an identical total loss. Real (**b**) and imaginary (**c**) parts of the eigenfrequencies (normalized in units of ${f}_{0}$ here) as functions of *ϵ* and *κ*, clearly exhibiting the broken phase (*ϵ* < *κ*) and the symmetry phase (*ϵ* > *κ*), which are divided by the exceptional line (*ϵ* = *κ*)$.$ **d** Measured reflection spectra revealing eigenfrequency splitting near the EP as *R* increases, in agreement with the theoretical predictions. **e** Temperature-dependent evolution of the detuning $\epsilon$ and coupling strength $\kappa$ as functions of $R$ and initial temperature ${T}_{0},$ in which the intersection of two planes indicates the exceptional line. **f** Heatmap of eigenfrequency splitting $\Delta f$ as a function of system parameters $R$ and ${T}_{0},$ similarly illustrating the exceptional line where $\Delta f=0.$ The presence of this exceptional line is independent of mismatches between PZT elements

Within the framework of a coupled-resonator structure, the PT symmetry requires the Hamiltonian $$({H}^{{PT}})$$ of the system to satisfy $$[{PT},{H}^{{PT}}]=0,$$ where *P* and *T* are parity and time-reversal operators, respectively. Such symmetry is typically realized by introducing balanced gain and loss into two oscillators coupled through a lossless energy interaction between them^55^. On the other hand, the APT symmetry simply yields $${{H}^{{APT}}={iH}}^{{PT}}$$ and $$\{{PT},{H}^{{APT}}\}=0,$$ which does not mandatorily require the implementation of a gain element, in general. While one possible circuit realization of such an APT-symmetric system has been discussed in ref. ^45^, here we propose a new schematic to better fit the PZT implementation, as shown in Fig. 2a. Two PZTs $$($$*X*_1_ and *X*_2_$$)$$ serve as two resonators that can be equivalent to a series connection of $${R}_{\mathrm{1,2}},{C}_{\mathrm{1,2}}$$, and $${L}_{\mathrm{1,2}}$$ using the Butterworth-Van Dyke (BVD) model^56^. Note that the complete BVD model refers to Supplementary Fig. S2b in Supplementary Note II, with an additional static capacitance $${C}_{0}$$ paralleled with those series-wound components. Without loss of generality, $${C}_{0}$$ is omitted as it produces no difference in the following analysis. Notably, the present theoretical analysis is based on the BVD equivalent models of both PZT resonators. Because the BVD framework is accurate only near the resonance frequency, the two PZTs should exhibit only limited intrinsic frequency detuning. If the detuning becomes too large, the operating point may fall outside the range over which the model remains valid, thus reducing the rigor and reliability of the analysis. In addition, excessive detuning would hinder effective coupling between the resonators. Since no two PZTs are exactly the same, $${L}_{1}{{\alpha }_{1}}^{-1}={L}_{2}{{\alpha }_{2}}^{-1}=L$$ and $${C}_{1}{{\beta }_{1}}^{-1}={C}_{2}{{\beta }_{2}}^{-1}=C$$ are assumed here, and $${\omega }_{0}=2\pi {f}_{0}=1/\sqrt{{LC}}$$ $$({f}_{0}=210.919\,\mathrm{kHz}).$$ Additionally, $${R}_{s1}$$ and $${R}_{s2}$$ are exploited to ensure that the total loss in two resonators is the same (i.e., $${R}_{s1}+{R}_{1}={R}_{s2}+{R}_{2}=-{R}_{3}=R).$$ The specific parameters mentioned above are detailed in the Method.

Applying Kirchhoff’s law, we obtain the circuit dynamics (explicit expressions are in Supplementary Note I), and the corresponding 2 × 2 Hamiltonian in the frame of CMT is

1 $${H}^{{APT}}=\left(\begin{array}{ll}-\epsilon & i\kappa \\ i\kappa & \epsilon \end{array}\right)$$

where $$\epsilon =({\omega }_{1}-{\omega }_{2})/2$$ is the angular resonance frequency detuning between two PZT resonators $$({\omega }_{1,2}=1/\sqrt{{\alpha }_{\mathrm{1,2}}{\beta }_{\mathrm{1,2}}}$$, in units of *ω*_0_$$)$$ and $$\kappa =R{(2\sqrt{{\alpha }_{1}{\alpha }_{2}}{L\omega }_{0})}^{-1}$$ denotes the coupling strength between them. One can then readily verify the APT symmetry by $$\{{PT},{H}^{{APT}}\}=0.$$ The eigenvalues of this non-Hermitian Hamiltonian simply read

2 $${\lambda }_{\pm }=\pm \sqrt{{\epsilon }^{2}-{\kappa }^{2}}$$

The eigenfrequencies of this resonator system are thus ${f}_{\pm }={(2\pi )}^{-1}{\omega }_{0}(s+{\lambda }_{\pm })={f}_{0}(s+{\lambda }_{\pm }),$ whose real part corresponds to the resonance-dip in the measured spectral profiles^45^; here, $s=({\omega }_{1}+{\omega }_{2})/2$ represents the average uncoupled-resonance angular frequency. Inspecting Eq. (2), the eigenfrequencies behave as a complex conjugate pair when $\epsilon < \kappa ,$ and become two purely real branches for $\epsilon > \kappa$ (Fig. 2b, c). These two regions correspond to the broken phase and the symmetry phase, and the boundary between them is the so-called EP $(\epsilon =\kappa ).$ In practice, we probe these eigenmodes by connecting this isolated system to an input/output channel, which, in the radio frequency (RF) and microwave region, is a vector network analyzer (VNA, red part in Fig. 2a). Note here that the participation of this terminal alters the effective coupling between two resonators, such that ${R}_{3}={R}_{s3}-{Z}_{0}$ is a mandatory correction for APT symmetry where ${Z}_{0}$ is the port impedance. The sign of this resistive coupling is then determined by ${R}_{s3}-{Z}_{0},$ i.e., ${R}_{s3} > {Z}_{0}$ denotes a lossy coupling, while ${R}_{s3} < {Z}_{0}$ corresponds to the coupling with gain. To avoid the imaginary part of eigenfrequencies, ${R}_{3}=-R < 0$ is selected as detailed in Supplementary Note VIII. With this setup, reflection spectra measured by the VNA (Fig. 2d) clearly show the eigenfrequency bifurcation $(\Delta f={f}_{0}\Delta \lambda ,\Delta \lambda ={\lambda }_{+}-{\lambda }_{-}):$ around the EP, the decrease of $R$ makes two eigenfrequencies bifurcate to two purely real branches, evident from the shifts of reflection dips (Fig. 2d).

In contrast to PT-symmetric systems, discrepancies between two PZTs do not break the APT symmetry since the diagonal elements in Eq. (1) depend solely on the frequency detuning, and as a result, the EP operation is always guaranteed. This characteristic not only enables a robust realization in practice using PZT resonators but also renders the reconfigurability of the proposed system. Specifically, owing to the temperature dependence of the material properties of the PZTs we used, the equivalent inductances ${L}_{\mathrm{1,2}}$ and capacitances ${C}_{\mathrm{1,2}}$ are nearly linearly proportional to the initial temperature ${T}_{0}$ over the operating range, whereas the equivalent resistances ${R}_{\mathrm{1,2}}$ remain almost constant (extracted from measurements in Supplementary Note II). As a result, the ${\alpha }_{\mathrm{1,2}}$ and ${\beta }_{\mathrm{1,2}}$ are temperature-relevant parameters, which yield the same relationship for $\epsilon$ and $\kappa$ as plotted in Fig. 2e. That is said, for different ${T}_{0}$ in various sensing scenarios, one can always reconfigure the system by tuning *R* (the method of tuning *R* is detailed in Supplementary Note IV) to find the intersection of the $\epsilon$ and $\kappa$ planes in Fig. 2e (indicating the exceptional line), and therefore, operate the system again at the EP to benefit from the sensitivity enhancement. This reconfigurability can also be observed from the heatmap of the frequency splitting $\Delta f$ in Fig. 2f, which accordingly allows us to reconfigure the system with an initial splitting of $\Delta f=0$ (EP operation). We emphasize that this property is not achievable in conventional PT-symmetric structures.

As a side note, we should emphasize that a positive temperature perturbation may drag the system operating from symmetry phase to the broken phase (Fig. 2f). Therefore, the initial temperature ${T}_{0}$ should always be configured slightly above the target temperature to ensure that the system operates within the symmetry phase.

### Temperature sensing based on APT-EP

After establishing the APT-symmetric structure using two PZTs, we then evaluate its capabilities for temperature sensing. The system is first tuned to operate at its EP at an initial temperature ${T}_{0},$ and then a small temperature perturbation $\Delta T={T}^{{\prime} }-{T}_{0}$ is introduced. Note here that $\Delta T < 0$ is required to ensure the system operates within the symmetry phase. As discussed previously, the frequency detuning and coupling strength between two PZTs are both linear functions of temperature as $\epsilon ={a}_{1}T+{b}_{1}$ and $\kappa =R({a}_{2}T+{b}_{2})$ (details of ${a}_{\mathrm{1,2}}$ and ${b}_{\mathrm{1,2}}$ are included in Supplementary Note II). Hence, with the normalized perturbation, $\delta =|\Delta {T|}/{T}_{0},$ the perturbed Hamiltonian reads

3 $${H}^{{\prime} }={H}_{{T}_{0}}^{{APT}}-\delta {T}_{0}\left(\begin{array}{cc}-{a}_{1} & i{a}_{2}R\\ i{a}_{2}R & {a}_{1}\end{array}\right),$$

where ${H}_{{T}_{0}}^{{APT}}$ denotes the Hamiltonian at ${T}_{0}.$ As expected, a small temperature variation will alter both the frequency detuning and coupling strength between two PZTs, and therefore, the associated eigenvalues under such perturbation are

4 $${{\lambda }_{\pm }}^{{\prime} }=\pm \sqrt{{\left({\epsilon }_{{T}_{0}}-\delta {T}_{0}{a}_{1}\right)}^{2}-{\left({\kappa }_{{T}_{0}}-\delta {T}_{0}R{a}_{2}\right)}^{2}}$$

Here, ${\epsilon }_{{T}_{0}}$ and ${\kappa }_{{T}_{0}}$ are fixed system parameters at the initial temperature ${T}_{0}.$ It is then direct to see that by adjusting the system parameters to invoke the EP operation $({\epsilon }_{{T}_{0}}={\kappa }_{{T}_{0}}),$ a small change will induce a drastic bifurcation of eigenfrequency that follows the square-root law as (detailed in Supplementary Note III)

5 $${\Delta f}^{{\prime} }={f}_{0}\Delta {\lambda }^{{\prime} }\approx 2{f}_{0}\sqrt{2{\epsilon }_{{T}_{0}}(R{a}_{2}-{a}_{1}){T}_{0}\delta }$$

serving as the foundation of the sensitivity enhancement of our temperature sensor.

**Fig. 3:**
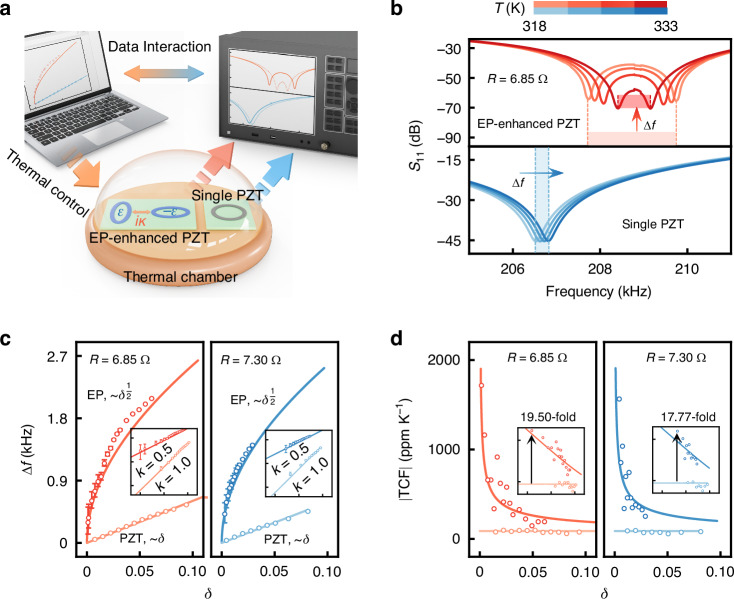
Experimental validation of temperature sensitivity enhancement by EP operation. **a** Experimental setup for temperature monitoring based on an EP-enhanced PZT sensing system and a reference single PZT resonator inside a programmable-controlled thermal chamber. Eigenfrequencies are extracted through reflection spectra measured using a VNA. **b** Measured reflection spectra for temperature variations at *R* = 6.85 Ω, showing strong eigenfrequency bifurcation (1.275 kHz) for the EP-enhanced system and markedly weaker frequency shifts (0.285 kHz) for the single PZT sensor. **c** Eigenfrequency splitting versus the normalized perturbation $\delta$ demonstrating clear square-root scaling for the EP-enhanced architecture (the slope $k=0.5$ in log-scale insets) and a linear response for the single PZT control $(k=1.0),$ across two independently configured EP operating points. **d** TCF retrieved from experimental measurements at different initial temperatures, revealing up to the maximum of 19.50-fold and 17.77-fold improvement in TCF (marked in log-scale inset) compared with the single PZT sensor at $R=6.85\,\Omega \,({T}_{0}=335.5\mathrm{K})$ and $R=7.30\,\Omega \,({T}_{0}=324.4\mathrm{K})$ respectively, validating the substantial performance boost provided by the APT-EP mechanism

To highlight such enhancements brought by the EP operation, we compare it with a conventional PZT temperature sensor, with the experimental setup illustrated in Fig. 3a. The EP-enhanced PZT temperature sensor (blue) and a single PZT sensor (gray) are placed within a thermal chamber, and their eigenfrequencies, embedded in the reflection spectra and probed by the VNA, are sent to an intelligent terminal (e.g., laptop) for programmably controlling the temperature of the chamber. Note that the EP-enhanced PZT sensor and the single PZT sensor here are temporarily composed of the PZTs ${X}_{1}$ and ${X}_{2}$ via fixtures (detailed in the Supplementary Note VI). After this experiment, the PZTs are removed for the fabrication of the EP-enhanced PZT sensor, referring to Supplementary Fig. S6a in Supplementary Note IV. The measured reflection spectra are plotted in Fig. 3b, in which the initial temperature is ${T}_{0}=335.5\,K$ with $\Delta T$ swept from −2.5 K to −17.5 K while $R=6.85\,\Omega$ is fixed. After setting the parameter *R*, we put the two systems into the chamber with thin cables through the connectors at the bottom of the chamber for the connection to the VNA. One can clearly observe two sharp resonance-dips exhibited by the APT-symmetric architecture, which experience a strong bifurcation (1.275 kHz). In stark contrast, the resonance shift of the single PZT is limited (0.285 kHz), clearly demonstrating the longstanding sensitivity challenge of conventional PZT temperature sensors.

A more straightforward comparison of the sensitivity of two systems can be observed from Fig. 3c, which plots the eigenfrequency bifurcation versus the normalized perturbation $\delta .$ A clear square-root scaling well confirms the enhanced sensitivity brought by the EP operation: in the small perturbation limit $(\delta \ll 1),$ this EP-enhanced system significantly outperforms the linear response given by the single PZT sensor. In parallel to this sensitivity evaluation, we also validate the reconfigurability of our system by setting a new initial temperature in the thermal chamber to ${T}_{0}=324.4\,K.$ Because of the temperature variation, the elastic constants, density, and permittivity of PZTs change and bring the variation of their equivalent ${L}_{\mathrm{1,2}}$ and ${C}_{\mathrm{1,2}},$ thus it influences the ${\alpha }_{\mathrm{1,2}}$ and ${\beta }_{\mathrm{1,2}},$ and simultaneously changes the $\epsilon$ and $\kappa ,$ as shown in Fig. 2e. Consequently, the system is removed from the chamber and allowed to cool sufficiently. It is then reconfigured to a new EP by adjusting $R=7.30\,\Omega$ along the exceptional line shown in Fig. 2f, after which it is placed back into the chamber. Applying the same temperature perturbations, the system exhibits exactly the same sensitivity as can be seen in the right panel of Fig. 3c. Taking into account the well-defined TCF, which is generally used to evaluate the sensitivity of resonator-based and frequency-based temperature sensors, we can quantitatively evaluate how much the sensitivity is augmented by the APT-EP. The TCF is expressed as

6 $${\rm{TCF}}=\frac{1}{{f}_{+}}\frac{\partial {f}_{+}^{{\prime} }}{\partial {T}^{{\prime} }}-\frac{1}{{f}_{-}}\frac{\partial {f}_{-}^{{\prime} }}{\partial {T}^{{\prime} }}\approx -\frac{1}{{f}_{0}}\frac{\partial \Delta {f}^{{\prime} }}{{T}_{0}\partial \delta }=-\sqrt{\frac{2(R{a}_{2}-{a}_{1}){\epsilon }_{{T}_{0}}}{{T}_{0}\delta }}$$

with curves and data depicted in Fig. 3d. Note that the TCF of this EP-enhanced PZT sensor is actually induced by the total variation of both eigenfrequencies ${f}_{+}$ and ${f}_{-},$ clearly depicted in Fig. 3b. As evident from Fig. 3d, the EP operation brings a tremendous enhancement to the conventional PZT temperature sensor for its sensitivity. Specifically, a 19.50-fold improvement of the TCF is achieved at an initial temperature of ${T}_{0}=335.5\mathrm{K},$ as shown in the left panel of Fig. 3d, and a 17.77-fold enhancement of the TCF emerges at 324.4 K in the right panel. Together, these results unambiguously demonstrate the pronounced sensitivity amplification enabled by the APT-EP structure.

Interestingly, this TCF may become zero once ${a}_{1}=R{a}_{2},$ which makes the square-root term in Eq. (5) identically vanishing. In other words, this condition may lock the system along the exceptional line in Fig. 2e, f, so that no perturbation can drag the system away from it. Fortunately, this condition can be avoided in general and we can always benefit from a large TCF in the small perturbation limit from the analysis in Supplementary Note III. Consequently, the EP operation endows the PZT temperature sensing system with ultrahigh sensitivity toward tiny perturbations, which is especially critical for sub-0.1 K sensing; meanwhile, the APT-symmetric architecture brings reconfigurability, which is crucial for practical implementations for various scenarios.

**Fig. 4:**
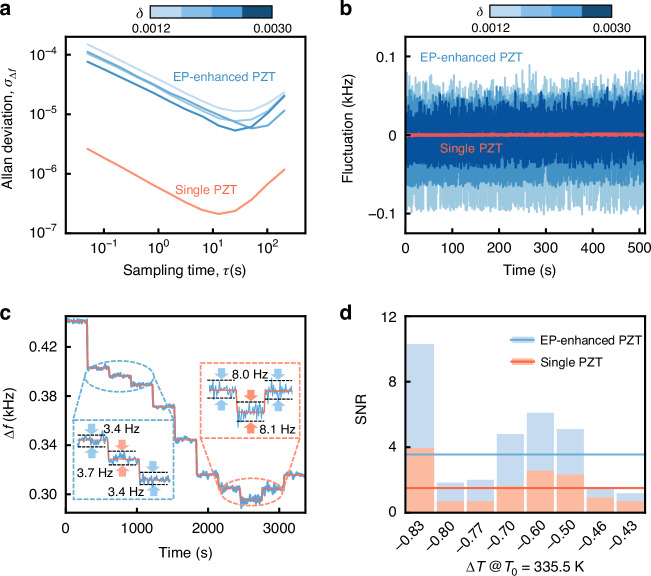
Noise robustness of the EP-enhanced PZT temperature sensor. **a** Allan deviation of the eigenfrequency splitting for small perturbations $(\delta =0.0012 \sim 0.0030),$ showing excellent frequency stability and confirming that the EP-enhanced system remains robust even under amplified noise conditions. **b** Time-domain frequency fluctuations measured over 500 s for both the EP-enhanced and single PZT sensors, confirming noise amplification near the EP. **c** Comparison between noise-induced fluctuations and signal-induced frequency steps for ultra-small temperature intervals (as low as 0.03 K). The blue line and orange line here represent the original and averaged frequencies, respectively, demonstrating that signal amplitudes exceed noise levels and enabling the LOD of the EP-enhanced PZT sensor down to 0.03 K. The sample size *n* = 3. **d** SNR extracted from frequency step measurements, with the blue and orange lines representing the averaged SNR of EP-enhanced PZT sensor (3.56) and single PZT sensor (1.51) respectively, showing that the EP-enhanced system exhibits significantly higher SNR, confirming excellent practicality in temperature sensing scenarios

In addition to the sensitivity enhancement, we next demonstrate the great noise robustness given by the proposed system, which yields an enhanced signal-to-noise ratio (SNR) compared to the conventional single PZT sensor. Typically, in this electronic system, the noise can be sourced from thermal noise, shot noise, and flicker noise, etc. Shot noise is generally observed in semiconductor devices and flicker noise is more pervasive at low frequencies (<1kHz) due to the $1/f$ characteristic of its power spectral density (PSD); consequently, the thermal noise dominates in our sensing system. The resilience against this thermal noise of a system associated with frequency manipulation can be evaluated by the frequency stability, which distinguishes the shifts induced by signals and fluctuations induced by unwanted noise. In line with this, we exploit the widely used statistical metric, Allan deviation^54,57,58^, to quantitatively characterize the short-term frequency stability, which measures the frequency fluctuations over different time intervals. Generically, the Allan deviation of the eigenfrequency splitting reads

7 $${\sigma }_{\Delta f}(\tau )=\frac{1}{{f}_{0}}\sqrt{\frac{1}{2(M-1)}{\sum }_{n=1}^{M-1}{\left(\bar{\Delta {f}_{n+1}^{{\prime} }}-\bar{\Delta {f}_{n}^{{\prime} }}\right)}^{2}}$$

where *M* denotes the total number of measurements, $\tau$ is the sampling time, $\bar{\Delta {f}_{n}^{{\prime} }}$ represents the average eigenfrequency splitting for the EP-enhanced PZT sensor acquired during the sampling time interval $\left[n\tau ,\left(n+1\right)\tau \right].$ During the measurements of Allan deviation, we set the system parameter to *R* = 6.85 Ω$,$ which corresponds to an initial temperature *T*_0_ = 335.5 K, and place the sensor inside a thermal chamber. The temperature inside the thermal chamber is varied from 335.1 K to 334.5 K in steps of 0.2 K to provide different temperature perturbations $\delta$ ranging from 0.0012 to 0.0030. At each specific $\delta ,$ the corresponding dynamic spectrum is acquired using the VNA, comprising 5001 points spanning the 206–211 kHz range; concurrently, a MATLAB program is employed to extract the resonance-dips (eigenfrequencies) from the VNA spectra 10,241 times over a total sampling period of 512 s (indicating that *τ*_min_ = 0.05 s). These extracted values are subsequently used to calculate the average eigenfrequency splitting $\bar{{\Delta f}_{n}^{{\prime} }},$ from which the Allan deviation is ultimately derived. It is direct to observe from Fig. 4a that the Allan deviation remains outstandingly low in the small perturbation region $(\delta \in \left[0.0012,\,0.0030\right]),$ reaching the minimum of $5.33\times 1{0}^{-6}$ at $\delta =0.0030.$ This clearly indicates that our system exhibits great noise resilience. We should point out here that the Allan deviation tends to be smaller for a larger perturbation, which complies with the theoretical analysis in refs. ^57,58^: in the immediate vicinity of the EP, the inherent collapse of the eigenbasis triggers a significant amplification of stochastic noise. Fundamentally, this phenomenon is governed by the strong non-orthogonality of the coalescing eigenvectors in non-Hermitian systems, which is quantitatively described by the Petermann factor^59,60^. This phenomenon compromises the frequency stability of the system, manifested as an elevated Allan deviation. Conversely, as the temperature perturbation scales, the system undergoes a transition away from the EP region; this migration suppresses the noise-enhancement effect, thereby restoring frequency stability and yielding a characteristic reduction in the Allan deviation. However, we will show that this does not deteriorate the SNR $(=\Delta {f}^{{\prime} }/{f}_{0}{\sigma }_{\Delta f})$ of our system in the following. Although the Allan deviation of a single PZT sensor is considerably lower than that of the EP-enhanced PZT sensor because of a more direct and simpler signal transduction pathway operation; it does not yield a better SNR due to its poor sensitivity.

This result can be more straightforwardly understood from the direct measurements of frequency fluctuations in Fig. 4b. The random thermal agitations of the electrons in any conductive elements within our system may cause random frequency fluctuations around the center frequency (actually the eigenfrequency here), and are recorded in Fig. 4b over 500 s. It is much more clearly demonstrated that as the system’s operating point approaches the EP, the noise level increases significantly. Using the Allan deviation, the measured data are grouped into blocks of 512 points and averaged, and the maximum frequency fluctuation caused by noise is found to be 8.1 Hz $(\delta =0.0012),$ which is sufficiently low to maintain a high SNR. In Fig. 4c, after setting a temperature interval and removing the thermal equilibrium time window of the system, the plot further compares the signal-induced frequency shift and the noise-induced frequency fluctuations of the EP-enhanced PZT sensor. The two smallest signal-induced frequency shifts, averaging 6.7 Hz and 9.3 Hz, as induced by the minute temperature variation of 0.03 K applied at two temperatures (334.70 K and 335.07 K), which remain significantly larger than the noise-induced fluctuations of 3.50 Hz and 8.05 Hz, respectively. This result demonstrates that the EP-enhanced PZT sensor has a LOD down to 0.03 K, which is significantly smaller than that of the conventional single PZT temperature sensor (0.054 K). The combined results of noise performance and sensitivity enhancements yield an improved SNR, given by the ratio between eigenfrequency bifurcation and noise-induced fluctuations (detailed in Method). The corresponding results are plotted in Fig. 4d, demonstrating that, compared with the temperature sensor based on a single PZT (with an average $\bar{\mathrm{SNR}}=1.51),$ the EP operation $(\bar{\mathrm{SNR}}=3.56)$ enhances not only the sensitivity but also the SNR. Upon setting a new initial temperature ${T}_{0}=324.4\mathrm{K}$ and reconfiguring the system back to the EP operation, this enhancement is still valid, demonstrating its capability and feasibility to be implemented into various sensing scenarios. The detailed measurement procedures refer to the Supplementary Note V.

### Real-time temperature monitoring

Following the theoretical analysis and experimental validation of sensitivity and resolution, we further assess the practical performance of the EP-enhanced PZT resonant sensor in two representative temperature-monitoring scenarios. These experiments are designed to emulate typical low- and high-temperature environments encountered in biomedical and electronic systems, thereby demonstrating the versatility of the proposed platform. Benefiting from its high temperature sensitivity, superior resolution, and tunable wide measurement range (reconfigurability), the system is well-suited for applications requiring high-precision temperature control, such as biomedical incubation, electromechanical thermal regulation, and battery thermal management.

**Fig. 5:**
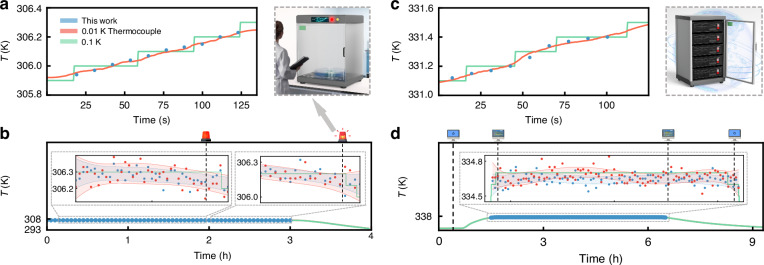
Real-time temperature monitoring in biological and high-temperature environments. **a** Real-time temperature monitoring compared with commercial thermometers of 0.1 K (green lines) and 0.01 K (red lines and dots) resolution, showing stable and accurate tracking by the EP-enhanced system (blue lines and dots). The EP-enhanced PZT sensor represented by a green square is inside a biological incubator (306.3 K), with minimal structural interference to the native temperature regulation. **b** Long-term stability test inside the incubator, demonstrating sustained sensing accuracy and smaller temperature fluctuations than those of a high-precision thermocouple. **c** Short-term thermal response comparison among EP-enhanced sensing, a standard thermocouple, and a high-precision reference thermometer. The green square inside the network server cabinet (334.7 K) is the EP-enhanced PZT sensor. **d** Long-term temperature trace in the server environment, showing that the EP-enhanced sensor maintains sub-0.1 K resolution with deviations within 0.03 K of a reference with 0.01 K accuracy, confirming its high stability and broad operational capability

The first scenario focused on low-temperature, high-stability control in a biological incubator $$(T{\rm{\approx }}33.3\,{\rm{^\circ }}{\rm{C}},306.3{\rm{K}}),$$ as depicted in Fig. 5a, b. Such incubators are widely used in cell culture and biomedical experiments, where even small temperature drifts can significantly affect biological activity and experimental reproducibility. To minimally disturb the existing setup, the EP-enhanced resonant sensing module is placed near the incubator door without noticeable structural modification (the green square inside the door in the inset of Fig. 5a), and $${T}_{0}=306.4\mathrm{K}$$ is configured. The incubator itself provides a nominal temperature control precision of 0.1 K. For benchmarking, reference temperatures are simultaneously recorded using two reference thermometers with specified accuracy of 0.1 K and 0.01 K, while the resonance frequency of the EP-enhanced PZT sensor is continuously tracked via a VNA and acquired by a computer. Note that in this scenario, both sensors are synchronized to acquire and record temperature data at 180-second intervals. This specific sampling rate is selected because our EP-enhanced sensor fundamentally captures real-time resonance frequency data, a brief processing window is required for the system to accurately compute and convert the raw frequency data into the final temperature readings. The monitored temperature in time traces (Fig. 5a) reveals that the sensor faithfully captures the subtle temperature variations within the chamber. In addition, a long-term monitoring experiment is conducted to evaluate stability (Fig. 5b). Over extended operation, the EP-enhanced PZT system maintains accurate and repeatable temperature readout of 306.23 ± 0.05 K, outperforming the reference thermocouple of 0.1 K in both resolution and sensitivity, and exhibiting a lower fluctuation compared with the reference thermocouple $$($$306.24 ± 0.07 K$$)$$ of 0.01 K resolution.

The second scenario targets a higher-temperature, thermally loaded environment: a network server equipment cabinet operating at ($T{\rm{\approx }}61.7\,{\rm{^\circ }}{\rm{C}},334.7{\rm{K}}$)$,$ as shown in Fig. 5c, d. In such systems, precise temperature monitoring is crucial for preventing thermal runaway, extending device lifetime, and enabling intelligent thermal management in data centers and communication infrastructures. Using a similar experimental procedure, the EP-enhanced resonant sensor with a selected ${T}_{0}=334.8\mathrm{K}$ is deployed inside the cabinet (the green square on the door in the inset of Fig. 5c) alongside the same thermocouple. In this scenario, we received a similar result to that of the biological incubators: the EP-enhanced PZT system has a much better sensitivity than the 0.1-K thermocouple and a more stable temperature readout (334.64 ± 0.04 K) than that of the reference thermocouple (334.67 ± 0.05 K) of 0.01 K. The continuous data acquisition is performed over an extended period of about 5 hours to thoroughly evaluate the long-term robustness and tracking accuracy of the system under typical server thermal loads. Both sensors are intentionally set to obtain temperature data at 180-second intervals here, either.

For further quantitative description of the high resolution, long-term stability, and robustness of the EP-enhanced PZT sensor, we calculated and recorded the mean values $(\bar{T})$ and standard deviations $(\mathrm{s.d.})$ of the temperature readouts exhibited by the EP-enhanced PZT sensor and the thermocouple separately in the two scenarios above. In the incubator scenario, the EP-enhanced PZT sensor and the thermocouple with an accuracy of 0.01 K recorded nearly identical $\bar{T}$ of 306.234 K and 306.240 K respectively, while our sensor has a smaller s.d. $=0.0698$ than that of the thermocouple with an s.d. = 0.0894$.$ Similar results are obtained in the server cabinet scenario: the EP-enhanced PZT sensor and the thermocouple yielded approximately equal $\bar{T}=334.643\mathrm{K}$ and $\bar{T}=334.669{\rm{K}}$, respectively, with a lower s.d. $=0.0408$ produced by our sensor than the s.d.$=0.0539$ produced by the thermocouple. These results provide convincing evidence for not only the high resolution but also the long-term stability and robustness of our sensing scheme under biologically relevant as well as high- and constant-temperature server cabinet conditions.

Together, both practical tests, spanning low-temperature biomedical incubation and high-temperature electronic equipment monitoring, highlight the broad applicability of the EP-enhanced PZT resonant platform. The demonstrated combination of high sensitivity, fine resolution, and stable performance across distinct environments underscores its potential as a universal, high-precision temperature sensing solution for real-world systems.

## Discussion

In conclusion, we have demonstrated an EP-enhanced piezoelectric thermometry platform that leverages APT symmetry to overcome the long-standing challenge of sensitivity in temperature sensing. By coupling two intrinsically mismatched PZT resonators through a lossy interaction, we realize a robust APT-symmetric architecture that can be reconfigured to operate at an EP across diverse temperature ranges. This configuration yields a TCF as high as −1500 ppm·K^−1^$,$ representing a 17-fold enhancement over the TCF of the conventional single PZT sensor and a 2-fold improvement over state-of-the-art resonant temperature sensors. Beyond static sensitivity, we have systematically evaluated the noise robustness and dynamic performance of the system. Allan deviation analysis and direct frequency-fluctuation measurements reveal that, despite noise amplification near the EP, the signal-induced eigenfrequency splitting consistently dominates noise-induced fluctuations, enabling an LOD down to 0.03 K and a marked enhancement in SNR compared to single PZT controls. Practical relevance is further established through real-time monitoring in two representative environments: a biomedical incubator and a thermally loaded server cabinet. The EP-enhanced PZT sensor delivers sub-0.1 K resolution and deviations within 0.03 K against a 0.01 K reference thermocouple in both cases, underscoring its accuracy, stability, and adaptability under realistic operating conditions.

More broadly, this work establishes a general paradigm for exploiting non-Hermitian physics in piezoelectric resonator sensing platforms. The combination of robustness against device mismatch, reconfigurability across operating conditions, and enhanced sensitivity suggests promising opportunities for extending this framework to wireless tags, distributed sensor networks, and multifunctional non-Hermitian electromechanical systems.

## Methods

### The equivalent BVD models of the PZT

In the experiments, *L* and *C* denote the dynamic inductance and dynamic capacitance of the PZT ${X}_{1}$ at the measured ambient temperature ${T}_{0}=300.9\mathrm{K},$ respectively, as defined by the BVD model. Because the APT-based EP-enhanced PZT sensor operates by exploiting temperature-induced variations in the eigenfrequency detuning *ϵ* and coupling coefficient $\kappa$ [described in Eqs. (4) and (5)] to modulate the eigenfrequency splitting, and because these quantities are derived from analyses of the BVD models of the two PZT elements, obtaining accurate BVD-equivalent parameters is essential. The BVD model can be extracted from the impedance profile of the PZT, which we acquire in the experiments using either an impedance analyzer or a VNA. Once the four key parameters characterizing the impedance curve are obtained, namely, the series resonant frequency ${f}_{s},$ the impedance ${Z}_{s}$ at ${f}_{s},$ the parallel resonant frequency ${f}_{p},$ and the impedance ${Z}_{p}$ at ${f}_{p},$ the individual component values of the BVD equivalent circuit can be calculated. The detailed derivation and formulas are provided in Supplementary Note II. The initial parameters *L* and *C*, representing the inductance and capacitance of the BVD model of PZT *X*_1_ at the measured ambient temperature ${T}_{0}=300.9\,\mathrm{K}$, are $L=241.47\,{\rm{\mu }}{\rm{H}}$ and $C=2.358\,{\rm{nF}}$, respectively.

### The measurement and calculation of LOD

Following the determination of the Allan deviation, we are able to evaluate the LOD of this system. The LOD at a given temperature is calculated by dividing three times the Allan deviation by the system’s sensitivity near that operating point, shown in the formula as $\mathrm{LOD}={3\sigma }_{\Delta f}/S$^61^. Here, *S* represents the normalized sensitivity of the system, written as $S=\partial \Delta {f}^{{\prime} }/{f}_{0}\partial {T}^{{\prime} }.$ As theoretically calculated, among the four temperatures tested (334.5 K, 334.7 K, 334.9 K, and 335.1 K) at ${T}_{0}=335.5\mathrm{K},$ the LOD at 335.1 K (nearest to the EP) is 0.022 K, whereas the lowest LOD of 0.015 K is achieved at 334.7 K. For a single PZT sensor, the experimental procedure of the measurement of the Allan deviation is virtually identical to that of the EP-enhanced device. The sole distinction is that we track the average resonance frequency $\bar{{f}_{{sn}}}$ rather than $\bar{{\Delta f}_{n}^{{\prime} }}.$ The Allan deviation ${\sigma }_{\Delta f}=2.11\times 1{0}^{-7}$ is extremely low, $S=8.8\times 1{0}^{-5}{\mathrm{K}}^{-1}.$ Therefore, the theoretical LOD is 0.007 K. However, the resolution of the VNA is 1 Hz, and the noise-induced frequency fluctuation, which is obtained by the procedure of grouping the measured data into blocks of 512 points and averaging, is 0.8 Hz. Consequently, resolving frequency shifts of approximately 0.13 Hz corresponding to a temperature variation of 0.007 K is neither feasible nor meaningful. Therefore, the practical frequency shift here is restricted to 1 Hz, which could be induced by a temperature variation of 0.054 K. As a result, the practical LOD of the PZT sensor is 0.054 K.

### The measurement and calculation of SNR

After obtaining the Allan deviation, the SNR could be estimated through $\mathrm{SNR}=\Delta {f}^{{\prime} }/{f}_{0}{\sigma }_{\Delta f}=\Delta {f}^{{\prime} }/\mathrm{AAD}$^62,63^. A small temperature variation of 0.03 K applied at a temperature of 335.07 K within a period of 2150.4 s to 3061.8 s (shown in Fig. 4c) results in a frequency shift $(\Delta {f}^{{\prime} })$ of 9.5 Hz of the EP-enhanced PZT system, and the AAD here could be regarded as a constant of 2.35 Hz (corresponding to $\delta =0.0012,$ referring to Fig. 4a); thus, the SNR is 4.25. Meanwhile, in practice, the SNR could also be directly obtained by the ratio of the step heights of the signal-induced frequency to the corresponding noise-induced fluctuations. In Fig. 4c, the step height is 9.5 Hz, and the average frequency noise of the upper (8.0 Hz) and lower (8.1 Hz) plates is 8.05 Hz, as a result, the practical SNR here is suppressed to 1.18. We record the practical SNR values computed using the latter method in Fig. 4d here. For the single PZT circuit, the SNR is likewise evaluated by the same procedure. Note that the theoretical AAD of this single PZT sensor is merely 0.045 Hz. When subjected to the same temperature variation of 0.03 K, it exhibits a theoretically estimated linear frequency shift of a mere 0.557 Hz, yielding a substantial theoretical SNR of 12.4. However, the experimentally measured average frequency noise of 0.8 Hz consequently suppresses the estimated SNR to a mere 0.696. Because the intrinsic frequency noise remains a low level across a wide temperature range, the technical noise dominates, and noise level can be treated as a constant 0.8 Hz across this region, and the effective SNR at other temperature deviations within this range can be reasonably and reliably estimated (as shown in Fig. 4d).

## Supplementary information

Supplementary information

## Data Availability

All data needed to evaluate the conclusions in the paper are present in the paper and/or the Supplementary Materials.

## References

[1] Cheong, J. et al. Fast detection of SARS-CoV-2 RNA via the integration of plasmonic thermocycling and fluorescence detection in a portable device. *Nat. Biomed. Eng.* **4**, 1159–1167 (2020). 33273713 10.1038/s41551-020-00654-0 PMC8202505

[2] Zhu, X. et al. Upconversion nanocomposite for programming combination cancer therapy by precise control of microscopic temperature. *Nat. Commun.* **9**, 2176 (2018). 29872036 10.1038/s41467-018-04571-4 PMC5988832

[3] Azuma, K. et al. Environmental factors involved in SARS-CoV-2 transmission: effect and role of indoor environmental quality in the strategy for COVID-19 infection control. *Environ. Health Prev. Med.* **25**, 66 (2020). 33143660 10.1186/s12199-020-00904-2 PMC7607900

[4] Li, C., Liu, Z., Goonetilleke, E. C. & Huang, X. Temperature-dependent kinetic pathways of heterogeneous ice nucleation competing between classical and non-classical nucleation. *Nat. Commun.* **12**, 4954 (2021). 34400646 10.1038/s41467-021-25267-2 PMC8367957

[5] Inoue, Y., Yokoyama, T., Yamasaki, N. & Tai, A. An optical yield that increases with temperature in a photochemically induced enantiomeric isomerization. *Nature* **341**, 225–226 (1989).

[6] Xu, X., Culligan, P. J. & Taylor, J. E. Energy saving alignment strategy: achieving energy efficiency in urban buildings by matching occupant temperature preferences with a building’s indoor thermal environment. *Appl. Energy* **123**, 209–219 (2014).

[7] Leng, F., Tan, C. M. & Pecht, M. Effect of temperature on the aging rate of li ion battery operating above room temperature. *Sci. Rep.* **5**, 12967 (2015). 26245922 10.1038/srep12967 PMC4526891

[8] Bhat, G., Gumussoy, S. & Ogras, U. Y. Power-temperature stability and safety analysis for multiprocessor systems. *ACM Trans. Embed. Comput. Syst.* **16**, 1–19 (2017).

[9] Nie, B. et al. Characterizing temperature, power, and soft-error behaviors in data center systems: insights, challenges, and opportunities. In *2017 IEEE 25th International Symposium on Modeling, Analysis, and Simulation of Computer and Telecommunication Systems (MASCOTS)* 22–31 (IEEE, 2017).

[10] Li, Q., Zhang, L., Tao, X. & Ding, X. Review of flexible temperature sensing networks for wearable physiological monitoring. *Adv. Healthcare Mater.* **6**, 1601371 (2017). 10.1002/adhm.201601371 28547895

[11] Kako, S. A comparative study about accuracy levels of resistance temperature detectors RTDs composed of platinum, copper, and nickel. *NJES* **26**, 216–225 (2023).

[12] Wagih, M. et al. Wide-range soft anisotropic thermistor with a direct wireless radio frequency interface. *Nat. Commun.* **15**, 452 (2024). 38199999 10.1038/s41467-024-44735-z PMC10781794

[13] Balčytis, A., Ryu, M., Juodkazis, S. & Morikawa, J. Micro-thermocouple on nano-membrane: thermometer for nanoscale measurements. *Sci. Rep.* **8**, 6324 (2018). 29679036 10.1038/s41598-018-24583-w PMC5910443

[14] Hong, L., Xiao, K., Song, X., Lin, L. & Xu, W. System-level modeling with temperature compensation for a CMOS-MEMS monolithic calorimetric flow sensing SoC. *Microsyst. Nanoeng.* **11**, 13 (2025). 39828749 10.1038/s41378-024-00853-8 PMC11743593

[15] Pathrose, J., Liu, C., Chai, K. T. C. & Xu, P. Y. A time-domain band-gap temperature sensor in SOI CMOS for high-temperature applications. *IEEE Trans. Circuits Syst. II* **62**, 436–440 (2015).

[16] Liang, X. et al. Temperature, pressure, and humidity SAW sensor based on coplanar integrated LGS. *Microsyst. Nanoeng.* **9**, 110 (2023). 37701521 10.1038/s41378-023-00586-0 PMC10493225

[17] Ahmed, Z. Assessing radiation hardness of silicon photonic sensors. *Sci. Rep.* **8**, 13007 (2018). 30158669 10.1038/s41598-018-31286-9 PMC6115432

[18] Gassino, R., Perrone, G. & Vallan, A. Temperature monitoring with fiber bragg grating sensors in nonuniform conditions. *IEEE Trans. Instrum. Meas.* **69**, 1336–1343 (2020).

[19] Hur, S., Mittapally, R., Yadlapalli, S., Reddy, P. & Meyhofer, E. Sub-nanowatt resolution direct calorimetry for probing real-time metabolic activity of individual C. elegans worms. *Nat. Commun.* **11**, 2983 (2020). 32532993 10.1038/s41467-020-16690-y PMC7293274

[20] Chen, H. et al. A two-stage amplified PZT sensor for monitoring lung and heart sounds in discharged pneumonia patients. *Microsyst. Nanoeng.* **7**, 55 (2021). 34567768 10.1038/s41378-021-00274-x PMC8433369

[21] Sui, W. et al. Micromachined thin film ceramic PZT multimode resonant temperature sensor. *IEEE Sens. J.* **24**, 7273–7283 (2024).

[22] Ilg, J., Rupitsch, S. J. & Lerch, R. Impedance-based temperature sensing with piezoceramic devices. *IEEE Sens. J.* **13**, 2442–2449 (2013).

[23] Yang, M., Zhu, L., Zhong, Q., El-Ganainy, R. & Chen, P.-Y. Spectral sensitivity near exceptional points as a resource for hardware encryption. *Nat. Commun.* **14**, 1145 (2023). 36854673 10.1038/s41467-023-36508-x PMC9974995

[24] Hodaei, H. et al. Enhanced sensitivity at higher-order exceptional points. *Nature* **548**, 187–191 (2017). 28796201 10.1038/nature23280

[25] Chen, W., Kaya Özdemir, Ş, Zhao, G., Wiersig, J. & Yang, L. Exceptional points enhance sensing in an optical microcavity. *Nature* **548**, 192–196 (2017). 28796206 10.1038/nature23281

[26] Uzdin, R., Dalla Torre, E. G., Kosloff, R. & Moiseyev, N. Effects of an exceptional point on the dynamics of a single particle in a time-dependent harmonic trap. *Phys. Rev. A* **88**, 022505 (2013).

[27] Khanbekyan, M. Exceptional-point-enhanced coupled microcavities for ultrasensitive particle sensing. *Phys. Rev. A* **108**, 023710 (2023).

[28] Wiersig, J. Enhancing the sensitivity of frequency and energy splitting detection by using exceptional points: application to microcavity sensors for single-particle detection. *Phys. Rev. Lett.* **112**, 203901 (2014).

[29] Lai, Y.-H., Lu, Y.-K., Suh, M.-G. & Vahala, K. Enhanced sensitivity operation of an optical gyroscope near an exceptional point. *Nature* **576**, 65–69 (2019). 31802018 10.1038/s41586-019-1777-z

[30] De Carlo, M., De Leonardis, F. & Passaro, V. M. N. Anti-PT-symmetric optical gyroscope at the transmission peak degeneracy with enhanced signal-to-noise ratio. *J. Lightwave Technol.* **42**, 936–944 (2024).

[31] Hokmabadi, M. P., Schumer, A., Christodoulides, D. N. & Khajavikhan, M. Non-hermitian ring laser gyroscopes with enhanced sagnac sensitivity. *Nature* **576**, 70–74 (2019). 31802015 10.1038/s41586-019-1780-4

[32] Zaky, Z. A., Hennache, A. & Zhaketov, V. D. Oral cancer biosensor using ternary photonic crystal and parity time symmetry. *Sci. Rep.* **15**, 33371 (2025). 41023050 10.1038/s41598-025-17739-y PMC12480628

[33] Li, J. et al. An ultrasensitive multimodal intracranial pressure biotelemetric system enabled by exceptional point and iontronics. *Nat. Commun.* **15**, 9557 (2024). 39500903 10.1038/s41467-024-53836-8 PMC11538422

[34] Ye, Z. et al. A highly sensitive and multiplexed wireless sensing system with skin-like compliance and stretchability for wearable applications. *Sci. Adv.* **11**, 4923 (2025). 10.1126/sciadv.adt4923 PMC12571053 41160676

[35] Özdemir, ŞK., Rotter, S., Nori, F. & Yang, L. Parity–time symmetry and exceptional points in photonics. *Nat. Mater.* **18**, 783–798 (2019). 30962555 10.1038/s41563-019-0304-9

[36] Lee, H. et al. Chiral exceptional point enhanced active tuning and nonreciprocity in micro-resonators. *Light Sci. Appl.* **14**, 45 (2025). 39788936 10.1038/s41377-024-01686-w PMC11718208

[37] Wang, C. et al. Electromagnetically induced transparency at a chiral exceptional point. *Nat. Phys.* **16**, 334–340 (2020).

[38] Shu, X. et al. Fast encirclement of an exceptional point for highly efficient and compact chiral mode converters. *Nat. Commun.* **13**, 2123 (2022). 35440654 10.1038/s41467-022-29777-5 PMC9018827

[39] Hu, L., Li, Y., Zhu, K., Chen, H. & Guo, Z. Linewidth narrowing and enhanced sensing in non-hermitian circuit systems via anti-PT symmetry. *Appl. Phys. Lett.* **126**, 091702 (2025).

[40] Yang, M. et al. Experimental observation of coherent-perfect-absorber and laser points in anti-PT symmetry. *Phys. Rev. A* **110**, 033504 (2024).

[41] Bergman, A. et al. Observation of anti-parity-time-symmetry, phase transitions and exceptional points in an optical fibre. *Nat. Commun.* **12**, 486 (2021). 33473141 10.1038/s41467-020-20797-7 PMC7817694

[42] Yang, Y. et al. Radiative anti-parity-time plasmonics. *Nat. Commun.* **13**, 7678 (2022). 36509769 10.1038/s41467-022-35447-3 PMC9744817

[43] Jahangiri, M., Parsanasab, G.-M. & Hajshahvaladi, L. Observation of anti-PT-symmetry and higher-order exceptional point PT-symmetry in ternary systems for single-mode operation. *Sci. Rep.* **15**, 4823 (2025). 39924509 10.1038/s41598-025-85623-w PMC11808060

[44] Yu, T., Liao, Z. & Liu, L. Observation of an anti-PT-symmetric exceptional point in plasmonics. In *2025 IEEE MTT-S International Wireless Symposium (IWS)* 1–3 (IEEE, 2025).

[45] Choi, Y., Hahn, C., Yoon, J. W. & Song, S. H. Observation of an anti-PT-symmetric exceptional point and energy-difference conserving dynamics in electrical circuit resonators. *Nat. Commun.* **9**, 2182 (2018). 29872042 10.1038/s41467-018-04690-y PMC5988699

[46] Zeng, Y.-W., Chen, W.-X., Yang, T.-L., Su, W.-J. & Wu, H. Sensing weak anharmonicities with a passive-active anti-PT symmetric system. *Phys. Rev. A* **113**, 023705 (2026).

[47] Huang, Y., Yu, A. & Xia, L. Anti-PT symmetric resonant sensors for nonreciprocal frequency shift demodulation. *Opt. Lett.* **50**, 3716 (2025). 40445689 10.1364/OL.562495

[48] Nair, J. M. P., Mukhopadhyay, D. & Agarwal, G. S. Enhanced sensing of weak anharmonicities through coherences in dissipatively coupled anti-PT symmetric systems. *Phys. Rev. Lett.* **126**, 180401 (2021). 34018771 10.1103/PhysRevLett.126.180401

[49] Campanella, H., Narducci, M., Merugu, S. & Singh, N. Dual MEMS resonator structure for temperature sensor applications. *IEEE Trans. Electron. Devices* **64**, 3368–3376 (2017).

[50] Moosavifar, M., Ansari, A. & Rais-Zadeh, M. An AlN-on-si resonant IR sensor array with a large temperature coefficient of frequency. In *2016 IEEE SENSORS* 1–3 (IEEE, 2016).

[51] Yuan, Y. et al. A piezoelectric MEMS resonant temperature sensor with 10-μk resolution and 0.06-PJK^2^ resolution FOM. In *2024 IEEE 37th International Conference on Micro Electro Mechanical Systems (MEMS)* 983–986 (IEEE, 2024).

[52] Zou, X., Ahmed, S., Jaber, N. & Fariborzi, H. A compact high-sensitivity temperature sensor using an encapsulated clamped-clamped mems beam resonator. In *2021 21st International Conference on Solid-State Sensors, Actuators and Microsystems (Transducers)* 1239–1242 (IEEE, 2021).

[53] Haus, H. A. & Huang, W. Coupled-mode theory. *Proc. IEEE* **79**, 1505–1518 (1991).

[54] Kononchuk, R., Cai, J., Ellis, F., Thevamaran, R. & Kottos, T. Exceptional-point-based accelerometers with enhanced signal-to-noise ratio. *Nature* **607**, 697–702 (2022). 35896648 10.1038/s41586-022-04904-w

[55] El-Ganainy, R. et al. Non-hermitian physics and PT symmetry. *Nat. Phys.* **14**, 11–19 (2018).

[56] Van Dyke, K. S. The piezo-electric resonator and its equivalent network. *Proc. IRE* **16**, 742–764 (1928).

[57] Suntharalingam, A., Fernández-Alcázar, L., Kononchuk, R. & Kottos, T. Noise resilient exceptional-point voltmeters enabled by oscillation quenching phenomena. *Nat. Commun.* **14**, 5515 (2023). 37679332 10.1038/s41467-023-41189-7 PMC10484910

[58] Giglio, M. et al. Allan deviation plot as a tool for quartz-enhanced photoacoustic sensors noise analysis. *IEEE Trans. Ultrason., Ferroelect. Freq. Contr.* **63**, 555–560 (2016). 10.1109/TUFFC.2015.2495013 26529758

[59] Chen, C., Jin, L. & Liu, R.-B. Sensitivity of parameter estimation near the exceptional point of a non-hermitian system. *New J. Phys.* **21**, 083002 (2019).

[60] Wang, H., Lai, Y.-H., Yuan, Z., Suh, M.-G. & Vahala, K. Petermann-factor sensitivity limit near an exceptional point in a brillouin ring laser gyroscope. *Nat. Commun.* **11**, 1610 (2020). 32235844 10.1038/s41467-020-15341-6 PMC7109037

[61] Shrivastava, A. & Gupta, V. Methods for the determination of limit of detection and limit of quantitation of the analytical methods. *Chron. Young Sci.* **2**, 21 (2011).

[62] Rife, D. & Boorstyn, R. Single tone parameter estimation from discrete-time observations. *IEEE Trans. Inf. Theory* **20**, 591–598 (2003).

[63] Quinn, B. G. & Kootsookos, P. J. Threshold behavior of the maximum likelihood estimator of frequency. *IEEE Trans. Signal Proces.* **42**, 3291–3294 (2002).

