# Supplementary material for: Exceptional point-enhanced piezoelectric thermometry via anti-parity−time symmetry
Source: Microsyst Nanoeng. 2026 Jun 15;12:234. doi: 10.1038/s41378-026-01353-7 (PMC13265762; doi:10.1038/s41378-026-01353-7)
Supplement: Supplementary file 1 — Supplementary information [file 41378_2026_1353_MOESM1_ESM.docx]

## Exceptional point-enhanced piezoelectric thermometry via anti-parity−time symmetry

## Supplementary Materials

Jiajun Wang^1†^, Jie Li^1†^, Bei Wu^1^, Xiwei Huang^1^, Wenjun Li^1^, Jikui Luo^2,3^, Minye Yang^4*^, Shurong Dong^2,*^, Weipeng Xuan^1*^

*^1^College of Electronics and Information, Ministry of Education Key Laboratory of RF Circuits and System, Hangzhou Dianzi University, Hangzhou, China*

*^2^College of Information Science and Electronic Engineering, Zhejiang University, Hangzhou 310027, China*

*^3^International Joint Innovation Center, Zhejiang University, Haining 314400, China*

*^4^School of Artificial Intelligence, Optics and Electronics, Northwestern Polytechnical University, Xi’an 710072, Shaanxi, China*

^†^These authors contributed equally to this work

*Corresponding Author: myang@nwpu.edu.cn; dongshurong@zju.edu.cn; xuanweipeng@hdu.edu.cn

**Supplementary Note I: Hamiltonian in coupled-mode theory**

The 2×2 Hamiltonian of the anti-parity–time (APT)-symmetric system, i.e., Eq. (1) in the main text, can be derived within the framework of coupled-mode theory (CMT). This approach enables direct comparison between electrical domain findings and other CMT-compatible systems, such as optical resonators. Detailed theoretical analysis of the CMT methodology is provided in References [1,2].

|  |
| --- |

Fig. S1. Simplified schematic diagram of APT-symmetric EP-enhanced PZT sensor, with the $\boldsymbol{L}_{\boldsymbol{1,2}}$ and $\boldsymbol{C}_{\boldsymbol{1,2}}$ extracted from the Butterworth-Van Dyke (BVD) model of lead zirconate titanate $\boldsymbol{(}\text{PbZ}\text{r}_{\boldsymbol{x}}\text{T}\text{i}_{\boldsymbol{1}\boldsymbol{-}\boldsymbol{x}}\boldsymbol{O}_{\boldsymbol{3}}\boldsymbol{,}$ PZT) $\boldsymbol{X}_{\boldsymbol{1,2}}\boldsymbol{,}$ and $\boldsymbol{R}_{\boldsymbol{1}}\boldsymbol{=}\boldsymbol{R}_{\boldsymbol{2}}\boldsymbol{=}\boldsymbol{-}\boldsymbol{R}_{\boldsymbol{3}}\boldsymbol{=R.}$

The circuit model of the APT-symmetric exceptional point (EP)-enhanced resonance sensor can be simplified to the schematic diagram shown in Fig. S1, where the two PZT resonators, $X_{1}$ and $X_{2}$ in the main text, are replaced by discrete components$C_{1},$ and$C_{2},$ exhibiting resonant angular frequencies of $\omega_{1}$ and $\omega_{2},$ respectively. To derive the CMT equation, we first define the complex oscillation modes of the left and right resonators as

$\begin{matrix} a_{1}=\sqrt{\frac{C_{1}}{2}}V_{1}-i\sqrt{\frac{L_{1}}{2}}I_{1}, \\ a_{2}=\sqrt{\frac{C_{2}}{2}}V_{2}-i\sqrt{\frac{L_{2}}{2}}I_{2}, \end{matrix}$

where $V_{n} (n=1,2)$ denotes the voltage across the capacitance $C_{n},$ $I_{n}$ represents the current flowing through the inductance $L_{n},$ $I_{r}$ represents the current flowing through the coupling resistance $R_{3},$ and $a_{n}$ denotes the complex mode amplitude with the time dependence $\exp(i\omega_{n}t)$ in each LC resonator. Note that due to the resistive coupling with the gain we applied in the system referring to the main text, the $R_{3}$ here is actually negative. Based on Eq. (1.1), $V_{n}$ and $I_{n}$ can be expressed in terms of $a_{n}$ as

$\begin{matrix} V_{1}=\frac{1}{\sqrt{2C_{1}}}(a_{1}+a_{1}^{*}), I_{1}=\frac{i}{\sqrt{2L_{1}}}(a_{1}-a_{1}^{*}), \\ V_{2}=\frac{1}{\sqrt{2C_{2}}}(a_{2}+a_{2}^{*}), I_{2}=\frac{i}{\sqrt{2L_{2}}}(a_{2}-a_{2}^{*}). \end{matrix}$

For the circuit depicted in Fig. S1, by applying Kirchhoff’s laws, an equation set can be derived as

$\left\{ \begin{aligned} &I_{n}=C_{n}\frac{d}{dt}V_{n}, \\ &I_{1}+I_{2}-I_{r}=0, \\ &R_{1}I_{1}+R_{3}I_{r}+L_{1}\frac{d}{dt}I_{1}+V_{1}=0, \\ &R_{2}I_{2}+R_{3}I_{r}+L_{2}\frac{d}{dt}I_{2}+V_{2}=0, \\ &R_{1}=R_{2}=-R_{3}=R. \end{aligned} \right.$

Substituting Eq. (1.2) into Eq. (1.3), we arrive at the expression

$\left\{ \begin{aligned} &\frac{d}{dt}a_{1}=\frac{i}{\sqrt{L_{1}C_{1}}}a_{1}-\frac{R_{1}+R_{3}}{2\sqrt{L_{1}L_{2}}}a_{1}-\frac{R_{3}}{2\sqrt{L_{1}L_{2}}}a_{2}, \\ &\frac{d}{dt}a_{2}=\frac{i}{\sqrt{L_{2}C_{2}}}a_{2}-\frac{R_{2}+R_{3}}{2\sqrt{L_{1}L_{2}}}a_{2}-\frac{R_{3}}{2\sqrt{L_{1}L_{2}}}a_{1}. \end{aligned} \right.$

The circuit parameters are defined as follows: $L_{1}{\alpha_{1}}^{-1}=L_{2}{\alpha_{2}}^{-1}=L, C_{1}{\beta_{1}}^{-1}=C_{2}{\beta_{2}}^{-1}=C.$ The parameters *L* and *C* are the inductance and capacitance of PZT $X_{1}$ under a specific isothermal condition $(T_{0}=300.9\text{ K}),$ with values of $L=241.47 \text{μ}\text{H}$and $C=2.358\text{ nF,}$ respectively. Given that the two resonators exhibit nearly identical resonant frequencies, we have $\alpha_{1}\beta_{1}\approx1$ and $\alpha_{2}\beta_{2}\approx1.$ The resistances satisfy $R_{1}=R_{2}=-R_{3}=R.$ The fundamental resonant frequency is given by $\omega_{0}=1/\sqrt{LC},$ which yields the resonance frequencies of $X_{1}$ and $X_{2}$ respectively are $\omega_{1}=\omega_{0}/\sqrt{\alpha_{1}\beta_{1}}$ ​and $\omega_{2}=\omega_{0}/\sqrt{\alpha_{2}\beta_{2}}.$ Furthermore, the coupling strength is defined as $k=R(2\sqrt{\alpha_{1}\alpha_{2}}L)^{-1}.$ Consequently, Eq. (1.4) can be reformulated as

$\left\{ \begin{matrix} i\frac{d}{dt}a_{1}=-\omega_{1}a_{1}+ika_{2}, \\ i\frac{d}{dt}a_{2}=-\omega_{2}a_{2}+ika_{1}. \end{matrix} \right.$

In Eq. (1.5), under the assumption of weak coupling $(k\ll\omega_{1,2})$ and small detuning $\left| \omega_{1}-\omega_{2} \right|\ll2(\omega_{1}+\omega_{2})^{-1},$ we rewrite the CMT to

$\tilde{a}_{n}=\sqrt{\frac{C_{n}}{2}}v_{n}-i\sqrt{\frac{L_{n}}{2}}i_{n},$

by introducing the slowly-varying complex-envelope function $v_{n}(t)$ and $i_{n}(t)$ as

$\begin{matrix} 2V_{n}(t)=v_{n}(t)exp(i\omega_{s}t)+v_{n}^{*}(t)exp(-i\omega_{s}t), \\ 2I_{n}(t)=i_{n}(t)exp(i\omega_{s}t)+i_{n}^{*}(t)exp(-i\omega_{s}t), \end{matrix}$

where $\omega_{s}=(\omega_{1}+\omega_{2})/2$ represents the *​*average uncoupled-resonance angular frequency. By substituting Eq. (1.6) into Eq. (1.5) and replacing $a_{n}$​ with its transformed counterpart $\tilde{a}_{n},$​​ we obtain the Schrödinger-like equation in the coupled-mode region as

$i\frac{d}{dt}\left( \begin{matrix} \tilde{a}_{1} \\ \tilde{a}_{2} \end{matrix} \right)=\omega_{0}\left( \begin{matrix} \frac{\omega_{2}-\omega_{1}}{2\omega_{0}} & i\frac{k}{\omega_{0}} \\ i\frac{k}{\omega_{0}} & \frac{\omega_{1}-\omega_{2}}{2\omega_{0}} \end{matrix} \right)\left( \begin{matrix} \tilde{a}_{1} \\ \tilde{a}_{2} \end{matrix} \right).$

Here, $\epsilon=(\omega_{1}-\omega_{2})/2\omega_{0}$ represents the normalized angular frequency detuning between the two resonators, in units of $\omega_{0}.$ With the normalized resistive coupling coefficient $\kappa=k/\omega_{0},$ ​we thus arrive at the final form of the normalized Hamiltonian, i.e., Eq. (1) in the main text:

$H^{APT}=\left( \begin{matrix} -\epsilon& i\kappa\\ i\kappa& \epsilon\end{matrix} \right).$

The eigenvalues of $H^{APT}$ are determined by solving the characteristic equation $\lambda I-H^{APT}=0,$ which defines the energy spectrum of the unperturbed Hamiltonian

$\lambda_{\pm}=\pm\sqrt{\epsilon^{2}-\kappa^{2}}.$

It is obvious that the EP appears when $\epsilon=\kappa.$ Owing to the frequency shift of $-s$​ introduced in the eigenfrequencies by the complex envelope transformation, the eigenfrequencies of the system are given by

$f_{\pm}=(2\pi)^{-1}\omega_{0}(s\pm\sqrt{\epsilon^{2}-\kappa^{2}})=f_{0}(s+\lambda_{\pm}).$

**Supplementary Note II: Temperature characteristics of resonators**

Our EP-enhanced PZT sensor works near the EP. Due to the temperature-dependent characteristic of both PZTs, the EP is determined by the initial temperature $T_{0}$ and the external resistance *R*, thus the exceptional line is formed, referring to Figs. 2e and 2f in the main text. As a result, it is critical to analyze the relationship between PZTs and the temperature (actually, the initial temperature $T_{0}$ here). The analysis of PZT resonators $X_{n}$ $(n=1,2)$ commonly employs the Butterworth-Van Dyke (BVD) equivalent circuit model, which simplifies the intricate electrical behavior of the resonator into a network of discrete components: a dynamic branch $(R_{n}, L_{n}, C_{n})$ in parallel with a static capacitance $({C_{0}}_{n}).$ Therefore, it enables us to facilitate a more tractable analysis of its resonant characteristics. Before extracting the BVD model parameters, the impedance spectrum of the PZT must be characterized. We measure the temperature-dependent impedance of the PZT resonators using the setup illustrated in Fig. S2a. Each PZT is mounted in a dedicated fixture and placed inside a temperature-controlled chamber. The simplified schematic of this setup is shown in Fig. S2b, where $Z_{t}$ represents the combined impedance of the fixture and cabling, and the $Z_{t}$ is connected in series with the PZT across the network analyzer ports. During measuring, $Z_{t}$ can be approximately regarded as a constant resistance of 0.55 Ω. The influence of $Z_{t}$ is computationally removed during data processing using the Advanced Design System (ADS) software to isolate the intrinsic PZT response.

|  |
| --- |

Fig. S2. a Experiment setup of the PZT impedance measurement. b The simplified schematic of the measuring circuit and the complete BVD model of PZTs. $\boldsymbol{Z}_{\boldsymbol{t}}$ here represents the equivalent resistance of the wires and fixture, and the value, almost independent from temperature, is 0.55 Ω. c The impedance curves of $\boldsymbol{X}_{\boldsymbol{1}}$ and $\boldsymbol{X}_{\boldsymbol{2}}\boldsymbol{,}$ and curves of the series $\boldsymbol{(}\boldsymbol{f}_{\text{s1,2}}\boldsymbol{,}$ d) and parallel $\boldsymbol{(}\boldsymbol{f}_{\text{p1,2}}\boldsymbol{,}$ e) resonance frequencies versus the initial temperature $\boldsymbol{T}_{\boldsymbol{0}}$ obtained from it, demonstrate higher sensitivity and linearity (18 Hz/K and 10 Hz/K) of $\boldsymbol{X}_{\boldsymbol{2}}$ than those of $\boldsymbol{X}_{\boldsymbol{1}}$ (−10 Hz/K and −7 Hz/K), and greater variation in both $\boldsymbol{f}_{\text{s1,2}}$ than $\boldsymbol{f}_{\text{p1,2}}\boldsymbol{.}$

During the experiment, the initial temperature $T_{0}$ is gradually increased. Once the temperature is set and unchanged, the impedance curves of PZTs are recorded. The results of impedance curves versus temperature $T_{0},$ plotted in Fig. S2c, exhibit a color gradient from light to dark, indicating the rising temperature. Notably, the two resonators display opposing trends with increasing temperature: the impedance curve of $X_{1}$ shifts toward lower frequencies, whereas that of $X_{2}$ shifts toward higher frequencies. However, the impedances in the series $(Z_{s1,2})$ and parallel $(Z_{p1,2})$ resonance points almost remain unchanged for both devices with values of 0.55 Ω, as shown in Fig. S2c. The extracted series resonance frequencies $f_{s1},$ $f_{s2}$ and parallel resonance frequencies $f_{p1},$ $f_{p2}$ are annotated in the figure. Comparative analysis reveals that under the same temperature variation, $X_{2}$ exhibits superior sensitivity and linearity in both series (18 Hz/K) and parallel resonance frequencies (10 Hz/K) than that of $X_{1}$ (−10 Hz/K and −7 Hz/K respectively). Furthermore, for $X_{2},$ the series resonance frequency $f_{s2}$ demonstrates greater thermal sensitivity than its parallel resonance frequency $f_{p2}.$ Given that the EP-enhanced PZT temperature-sensing system operates by detecting shifts in its resonance frequency, $X_{2}$ is selected for constructing the single PZT temperature sensor, with its resonance frequency serving as the metric for tracking temperature-induced variations.

Following the acquisition of the impedance spectrum of the PZT, the equivalent circuit parameters of the BVD model, namely, the dynamic resistance $R_{n} (n=1,2),$ dynamic inductance $L_{n},$ dynamic capacitance $C_{n},$ and static capacitance $C_{0n},$ are extracted by fitting the experimental data to the theoretical impedance expression of the model, which is given by^3^

$Z_{n}\left( \omega\right)=\frac{(1-\omega^{2}L_{n}C_{n})+j\omega R_{n}C_{n}}{-\omega^{2}R_{n}C_{n}C_{0n}+j\omega[(C_{n}+C_{0n})-\omega^{2}L_{n}C_{n}C_{0n}]}.$

Furthermore, it is established that the material loss of the PZT is negligibly small, implying that

$f_{r}\approx f_{min}\approx f_{s} ,f_{a}\approx f_{max}\approx f_{p}$

According to the IEEE standard on piezoelectricity^4,5^, the resonant frequency is denoted as $f_{r},$ and the anti-resonant frequency is defined as $f_{a};$ $f_{min}$ is defined as the frequency at the minimum impedance point while $f_{max}$ is the frequency at the maximum impedance point; $f_{s}$ is the frequency of maximum conductance, and $f_{p}$ is the frequency of maximum resistance. Under low-loss conditions, the minimum-impedance point on the impedance curve can thus be approximated as $f_{s},$ while the maximum-impedance point corresponds to $f_{p}.$ These two critical frequencies of the two PZTs, $X_{1}$ and $X_{2},$ applied in the system, can be calculated as follows^3^:

$f_{sn}=\frac{1}{2\pi\sqrt{L_{n}C_{n}}}, n=1,2,$

$f_{pn}=\frac{1}{2\pi\sqrt{L_{n}\frac{C_{0n}C_{n}}{C_{0n}+C_{n}}}}=f_{sn}\sqrt{1+\frac{C_{n}}{C_{0n}}}.$

By simultaneously solving the equation set of Eq. (2.1) ~ (2.4), we derive explicit analytical expressions for the parameters of the BVD model:

$C_{0n}=\sqrt{\frac{Z_{sn}^{2}(\omega_{pn}^{2}-\omega_{sn}^{2})+\sqrt{(2\omega_{pn}^{2}Z_{sn}Z_{pn})^{2}+Z_{sn}^{4}(\omega_{pn}^{2}-\omega_{sn}^{2})^{2}}}{2(\omega_{pn}^{2}Z_{sn}Z_{pn})^{2}}},$

$R_{n}=\sqrt{\frac{Z_{sn}^{2}}{1-(\omega_{sn}Z_{sn}C_{0n})^{2}}},$

$C_{n}=C_{0n}\left[ \left( \frac{\omega_{pn}}{\omega_{sn}} \right)^{2}-1 \right],$

$L_{n}=\frac{1}{\omega_{sn}^{2}C_{n}}.$

The acquisition of the complete impedance curve for the BVD model enables the determination of key parameters, including the series resonance frequency $f_{sn},$ the impedance at series resonance $Z_{sn},$ the parallel resonance frequency $f_{pn},$ and the impedance at parallel resonance $Z_{pn}.$ These parameters allow for the extraction of the BVD equivalent circuit. To validate the approach, we compare the experimentally measured impedance of the PZT $X_{2}$ with the fitted curve generated by the BVD model from Eqs. (2,5) ~ (2.8), as shown in Fig. S3a. The fitted curve exhibits excellent agreement with the experimental data, confirming the accuracy and reliability of this impedance-analysis methodology.

|  |
| --- |

Fig. S3. a The curve obtained from the BVD model with Eqs. (2.5)~(2.8) and the experimental impedance values (circles) of the PZT $\boldsymbol{X}_{\boldsymbol{2}}\boldsymbol{,}$ showing a high degree of fitting and exhibiting an excellent accuracy of the explicit analytical expressions for the BVD model. The fitted data (line) and the experimental values of the dynamic capacitance $\boldsymbol{C}_{\boldsymbol{1,2}}$ (b) and the dynamic inductance $\boldsymbol{L}_{\boldsymbol{1,2}}$ (c) versus initial temperature $\boldsymbol{T}_{\boldsymbol{0}}$ from their impedance curves in various temperatures (293 K ~ 353 K) and Eqs. (2.5) ~ (2.8). All four fitted lines show an excellent coefficient of determination $\boldsymbol{(}\boldsymbol{R}^{\boldsymbol{2}}\boldsymbol{>0.97)}$ within this temperature range.

Using Eq. (2.5) through Eq. (2.8), we obtain numerical values for the parameters of the BVD equivalent circuit. Since the dynamic capacitance $C_{n}(n=1, 2)$ and dynamic inductance $L_{n}$ are mainly utilized in the subsequent analysis, we focus our analysis on these two parameters. The extracted values of $C_{n}$ and $L_{n}$​ for both PZT resonators versus the initial temperature $T_{0}$ are presented graphically in Figs. S3b and S3c, respectively. As shown in these figures, all parameters exhibit a nearly linear relationship with temperature; subsequent linear fitting yielded satisfactory results, with a high coefficient of determination of $R^{2}=0.978$ for $C_{1},$ $R^{2}=0.995$ for $C_{2},$ $R^{2}=0.973$ for $L_{1},$ and $R^{2}=0.998$ for $L_{2},$ allowing us to determine the slopes and intercepts of the fitted curves, which are then used to express $C_{n}$ and $L_{n}$ as linear functions of $T_{0},$ which is of vital importance to the derivation of the expressions for $\epsilon$ and $\kappa$against $T_{0}.$

Now we have already derived the expressions that $\epsilon, \kappa,$ and $s$ against $\alpha_{1,2}$ and $\beta_{1,2}$ from the previous text, that is

$\epsilon=\frac{1}{2\sqrt{\alpha_{1}\beta_{1}}}-\frac{1}{2\sqrt{\alpha_{2}\beta_{2}}},$

$\kappa=\frac{R}{2\omega_{0}\sqrt{\alpha_{1}\alpha_{2}}L},$

$s=\frac{1}{2\sqrt{\alpha_{1}\beta_{1}}}+\frac{1}{2\sqrt{\alpha_{2}\beta_{2}}}.$

Additionally, it is evident that the $\alpha_{1,2}$ and $\beta_{1,2}$ are linear expressions against initial temperature $T_{0}$ according to Fig. S3b and S3c based on their definitions. Thus, let us consider the linear expressions of $\alpha_{1,2}$ and $\beta_{1,2}$ against $T_{0}$ as follows: $\alpha_{1, 2}=c_{1,2}T_{0}+d_{1,2}, \beta_{1, 2}=g_{1,2}T_{0}+h_{1,2}.$ From the legend of Fig. S3b and S3c, it is able to estimate that the parameters $\left| c_{1,2} \right|, \left| g_{1,2} \right|\ll10^{-4},$ and $d_{1,2}, h_{1,2}\approx1.$ After doing these, we can rewrite the expressions of $\epsilon, \kappa,$ and $s$ by taking these linear expressions into Eq. (2.9) ~ (2.11), and taking the first-order Taylor expansion at $T_{0}=323\text{ K},$ which is the middle of the interval of [293 K, 353 K], we get

$\epsilon=(A_{1}-A_{2})T_{0}+(B_{1}-B_{2})=a_{1}T_{0}+b_{1},$

$\kappa=R(DT_{0}+E)=R(a_{2}T_{0}+b_{2}),$

$s=(A_{1}+A_{2})T+(B_{1}+B_{2}),$

and in these expressions,

$\left\{ \begin{aligned} &A_{n}=\left. -\frac{2c_{n}g_{n}T+c_{n}h_{n}+d_{n}g_{n}}{4}(c_{n}g_{n}T^{2}+c_{n}h_{n}T+d_{n}g_{n}T+d_{n}h_{n})^{-\frac{3}{2}} \right|_{T=323}, n=1, 2, \\ &B_{n}=\left. \frac{1}{2}(c_{n}g_{n}T^{2}+c_{n}h_{n}T+d_{n}g_{n}T+d_{n}h_{n})^{-\frac{1}{2}} \right|_{T=323}-323A_{n}, \\ &D=\left. -\frac{2c_{1}c_{2}T+c_{1}d_{2}+d_{1}c_{2}}{4}(c_{1}c_{2}T^{2}+c_{1}d_{2}T+d_{1}c_{2}T+d_{1}d_{2})^{-\frac{3}{2}} \right|_{T=323}, \\ &E=\left. \frac{1}{2}(c_{1}c_{2}T^{2}+c_{1}d_{2}T+d_{1}c_{2}T+d_{1}d_{2})^{-\frac{1}{2}} \right|_{T=323}-323D. \end{aligned} \right.$

Here, the parameters $c_{1,2}, d_{1,2}, g_{1,2},$ and $h_{1,2}$ could be derived precisely, thus we can have the $a_{1,2}$ and $b_{1,2}$ in the expressions of $\epsilon$ and $\kappa$ against $T_{0}$ are $a_{1}=-5.6335\times10^{-5},$ $a_{2}=-2.9501\times10^{-7},$ $b_{1}=0.02818,$ and $b_{2}=0.00145$ respectively. Given that parameter $s$ is less critical than the parameters $\epsilon$ and $\kappa$ in governing the behavior of the system, and considering that the final expression for frequency splitting does not involve $s,$ we focus primarily on elucidating the roles of $\epsilon$ and $\kappa.$ For completeness, the expression for$s$is provided as follows:

$s=-2.8949\times10^{-5}T_{0}+0.9802.$

The resulting data of $\epsilon$ and $\kappa/R$ against $T_{0}$ and functional forms are presented in Figs. S4a and S4b. In these figures, the discrete circles represent values obtained directly from experimental values of $C_{n}$ and $L_{n}$ depicted in Figs. S3b and S3c, while the green solid lines in each figure represent the theoretical data calculated through Eq. (2.12) and (2.13). The circles and theoretical curves for $\epsilon$ and $\kappa/R$ against temperature $T_{0}$ show excellent agreement, with the maximum deviation of sub-0.5%. As a result, both parameters can be accurately represented by linear functions, leading to the convenience for subsequent analysis.

To enhance the agreement between our theoretical model and experimental observations, the parameters in the expression are fine-tuned, thus the values of $a_{1,2}$ and $b_{1,2}$ are ultimately determined as follows, respectively.

$\left\{ \begin{aligned} &a_{1}=-5.63456\times10^{-5}, \\ &a_{2}=-2.9502\times10^{-7}, \\ &b_{1}=0.02817, \\ &b_{2}=0.00145. \end{aligned} \right.$

It is evident, referring to Fig. 2e in the main text, that in the temperature range of [293 K, 353 K], the surfaces of $\epsilon$ and $\kappa$ have only one line of intersection, the so-called exceptional line. This implies that for any initial temperature $T_{0}$ within this range, there exists a corresponding parameter *R* of the system on this line that positions the system at the EP. At temperatures below $T_{0}$ $(T^{'}<T_{0}),$ it is clearly observable in the plot that the condition $\epsilon>\kappa$ is satisfied, indicating that the system resides in the symmetry phase; in contrast, when the system is located where it is above $T_{0},$ the system steps into the broken phase. Therefore, to ensure the system remains in the symmetry phase during temperature measurement (with *R* held constant), it is necessary to maintain the measured temperature $T^{'}$ below $T_{0}.$

Fig. S4. The theoretical curves (green line) and experimental values (circle) of $\boldsymbol{\epsilon}$ (a) and $\boldsymbol{\kappa/R}$ (b) versus $\boldsymbol{T}_{\boldsymbol{0}}\boldsymbol{,}$ respectively, showing the excellent agreement, and indicating the high accuracy of the linear approximation of Eqs. (2.12) and (2.13).

A brief analysis of Eqs. (2.12) and (2.13) reveals that $\epsilon$ is a parameter influenced solely by the initial temperature $T_{0},$ whereas $\kappa$ depends not only on $T_{0}$ but also on the resistance *R*. Since the condition for the system to operate at the EP in the temperature of $T_{0}$ requires $\epsilon_{T_{0}}=\kappa_{T_{0}}$ (here, $\epsilon_{T_{0}}$ and $\kappa_{T_{0}}$ are fixed parameters at the initial temperature $T_{0}),$ this implies that the parameter *R* must satisfy the relation under such conditions:

$R=\frac{a_{1}T_{0}+b_{1}}{a_{2}T_{0}+b_{2}}.$

This expression indicates that for each initial temperature $T_{0},$ there exists only one corresponding system parameter *R*. This relationship provides the theoretical basis for the reconfigurability of the EP of our EP-enhanced PZT sensor.

**Supplementary Note III: The frequency response of the system under temperature perturbation**

At the EP under an initial temperature $T_{0},$ the condition $\epsilon_{T_{0}}=\kappa_{T_{0}}$ holds. By simultaneously adapting Eq. (2.12) and (2.13) under this condition, we obtain the following expressions:

$\epsilon=\epsilon_{T_{0}}+a_{1}(T^{'}-T_{0}),$

$\kappa=\kappa_{T_{0}}+Ra_{2}(T^{'}-T_{0}).$

Here, we define $\Delta T=T^{'}-T_{0}$ as the temperature perturbation; it is important to emphasize that the frequency splitting of the system $\Delta f$ generates as the temperature $T^{'}<T_{0}.$ Consequently, a negative temperature change (Δ*T* < 0) is implied here. Now we define the normalized perturbation $\delta=\left| \Delta T \right|/T_{0},$ thus, $T^{'}=T_{0}+\Delta T=T_{0}(1-\delta).$ We now incorporate the temperature perturbation $\delta,$ as defined in the main text, which yields the perturbed Hamiltonian and its corresponding eigenvalues

$H'=\left( \begin{matrix} -(\epsilon_{T_{0}}-\delta T_{0}a_{1}) & i(\kappa_{T_{0}}-\delta T_{0}a_{2}R) \\ i(\kappa_{T_{0}}-\delta T_{0}a_{2}R) & \epsilon_{T_{0}}-\delta T_{0}a_{1} \end{matrix} \right)=H_{T_{0}}^{APT}-\delta T_{0}\left( \begin{matrix} -a_{1} & ia_{2}R \\ ia_{2}R & a_{1} \end{matrix} \right),$

${\lambda_{\pm}}^{'}=\pm\sqrt{(\epsilon_{T_{0}}-\delta T_{0}a_{1})^{2}-(\kappa_{T_{0}}-\delta T_{0}a_{2}R)^{2}}.$

Here, in the vicinity of the EP, the eigenfrequency splitting of the system is given by $\Delta f=f_{0}\Delta\lambda,$ $\Delta\lambda=\lambda_{+}-\lambda_{-}.$ Substitution of the condition $\epsilon_{T_{0}}=\kappa_{T_{0}}$ into this expression, thus yields

$\Delta f^{'}=2f_{0}\sqrt{\left( a_{1}^{2}-R^{2}a_{2}^{2} \right)T_{0}^{2}\delta^{2}+\left( 2Ra_{2}\epsilon_{T_{0}}-2a_{1}\epsilon_{T_{0}} \right)T_{0}\delta}.$

Given that $\delta\ll1$, and $\left| a_{1,2} \right|\ll1$, therefore, the higher-order terms in $\delta$ within the square root of Eq. (3.5) could be neglected. Thus, we arrive at the final expression presented as

$\Delta f^{'}\approx2f_{0}\sqrt{2\epsilon_{T_{0}}(Ra_{2}-a_{1})T_{0}}\sqrt{\delta}.$

Eq. (3.6) indicates that the EP-enhanced PZT system can produce a nonlinear response to small perturbations $\delta$ generated by temperature variation, and it also shows the relationship of eigenfrequency splitting $\Delta f^{'}$ between $\delta$ is square-root scaling $(\Delta f^{'}\propto\delta^{1/2}).$ This inference is consistent with the experimental results referring to Figs. 1b and 3c.

To intuitively demonstrate the sensitivity enhancement effect of the EP-enhanced PZT system, we introduce the concept of the coefficient of frequency (TCF). TCF is widely used to describe the sensitivity of temperature sensors that work based on frequency shifting, for example, the resonator temperature sensors. The definition of the TCF of the eigenfrequencies is

$\text{TC}\text{F}_{\pm}=\frac{1}{{f_{\pm}}^{'}}\frac{\partial{f_{\pm}}^{'}}{\partial T^{'}},$

where $f_{\pm}$ represent the eigenfrequencies of the system, and $T^{'}$ represents the temperature. Referring to the Figs. 2d and 3b, the variation trends of the two eigenfrequencies $f_{+}$ and $f_{-}$ are opposite. When the temperature perturbation increases, the $f_{+}$ decreases while the $f_{-}$ is increasing; under the combined effect of them, the eigenfrequency bifurcation varies more violently in a small perturbation, thus the TCF of the system can be considered as the superposition of $\text{TC}\text{F}_{+}$ and $\text{TC}\text{F}_{-},$ i.e.

$\mathrm{TCF}=\frac{1}{{f_{+}}^{'}}\frac{\partial{f_{+}}^{'}}{\partial T^{'}}-\frac{1}{{f_{-}}^{'}}\frac{\partial{f_{-}}^{'}}{\partial T^{'}}\approx-\frac{1}{f_{0}}\frac{\partial\Delta f^{'}}{T_{0}\partial\delta}\approx-\sqrt{\frac{2(Ra_{2}-a_{1})\epsilon_{T_{0}}}{T_{0}\delta}}.$

Eq. (3.8) indicates that the TCF of the system reaches infinity when $\delta=0$. Furthermore, these results demonstrate the remarkably high sensitivity of the system to minute temperature perturbations near the EP. Also, we can readily observe that the equation admits a possible zero, at $a_{1}=Ra_{2}.$ Once this condition is established, the TCF may vanish by the way. We next consider the zero. If the zero holds, we have $R=a_{1}/a_{2}$. We then plot the curve of *R* against $T_{0}$ through this equation and Eq. (2.18) in Fig. S5 respectively, and the former equation represents the relationship between *R* and $T_{0}$ under the condition that the system parameters located on EP holds, while the latter means $\text{TCF}=0.$ It is clear that the two curves have no intersections within the range of $T_{0}\in[293 K, 353 K].$ Thus, no condition would cause the extinction of TCF, and the effect of sensitivity enhancement produced by APT-EP structure will always work throughout the temperature sensing process.

Fig. S5. The exceptional line from Eq. (2.18) and the curve from equation $R=a_{1}/a_{2}\boldsymbol{,}$ demonstrating no intersections within the temperature range of [293 K, 353 K].

**Supplementary Note IV: Methods of tuning the APT-symmetric system to the EP**

Fig. S6a shows the EP-enhanced PZT device used in this study. The top layer of the printed circuit board (PCB) contains no components; components are concentrated on the bottom layer, including three adjustable surface-mount resistors (NIDEC ST-32EA), a sub-miniature version A (SMA) female connector, and a pair of pins for measuring the resistance of $R_{s3}.$ Featuring a low temperature coefficient of resistance (TCR) of ± 250 ppm/K, these surface-mount resistors provide excellent thermal stability, thereby restraining sensing errors induced by resistance variations. The PZTs are integrated within the PCB, which are clearly visible under backlit imaging. Two PZT circular thin plates, together with the three external adjustable resistors, constitute the APT-EP system. When the system meets with APT-symmetry, and is located at the symmetry phase, the curve of $S_{11}$ is depicted in Fig. S6b. The curve exhibits a pair of symmetric, downward-indented resonance peaks with nearly identical depths.

As stated in the main text, for each initial temperature $T_{0}$ to be monitored, we adjust the system parameter *R* to locate the operating point at the EP. The system parameter *R* here denotes the equivalent resistance of each of the three branches shown in Fig. S1a, with the condition that $R_{1}=R_{2}=-R_{3}=R,$ for maintaining APT symmetry. However, the experimental circuit board used (shown in Fig. S6a) is compact in size, and the resistors soldered onto it are also very small, posing challenges for the practical adjustment of the system parameter *R* to the target value previously mentioned. Here, we employ a Smith chart-based systematic tuning procedure in which the resistance of ${R_{s}}_{3}$ labeled in Fig. S6a of the PCB is measured through the measurement pins, and the curve on the Smith chart is observed to set the desired system parameter *R*.

We first derive the analytical expression for the reflection coefficient $S_{11}$ of the system when the system meets APT symmetry. As shown in Fig. 2a, the $S_{11}$ of the system writes

$S_{11}=\frac{-i\gamma\lambda-\frac{\left( \lambda^{2}\alpha_{1}\beta_{1}-i\gamma\lambda\beta_{1}-1 \right)\left( \lambda^{2}\alpha_{2}\beta_{2}-i\gamma\lambda\beta_{2}-1 \right)}{\lambda^{2}(\alpha_{1}+\alpha_{2})\beta_{1}\beta_{2}-2i\gamma\lambda\beta_{1}\beta_{2}-(\beta_{1}+\beta_{2})}}{-i(\gamma-2\zeta)\lambda-\frac{\left( \lambda^{2}\alpha_{1}\beta_{1}-i\gamma\lambda\beta_{1}-1 \right)\left( \lambda^{2}\alpha_{2}\beta_{2}-i\gamma\lambda\beta_{2}-1 \right)}{\lambda^{2}(\alpha_{1}+\alpha_{2})\beta_{1}\beta_{2}-2i\gamma\lambda\beta_{1}\beta_{2}-(\beta_{1}+\beta_{2})}}.$

Here, $\zeta=Z_{0}\sqrt{C/L}$ and $\gamma=R\sqrt{C/L},$ $Z_{0}$ is the impedance of the transmission line, usually it is 50 Ω. The Fig. S6b actually displays the system $S_{11}$ curve in decibels, i.e., $20\lg\left| S_{11} \right|.$ Upon differentiating with respect to time and evaluating $\partial\lg\left| S_{11} \right|/\partial\lambda,$ we obtain an expression that admits two minima within a certain frequency range, satisfying $\partial\lg\left| S_{11} \right|/\partial\lambda=0;$ these two minima correspond to the two eigenfrequencies of the EP-enhanced PZT sensor. We then analyze the trajectory of $S_{11}$ on the Smith chart; the Smith chart is a polar plot of the reflection coefficient. We express the reflection coefficient of the system in polar form as $S_{11}=\left| S_{11} \right|e^{i\theta},$ plot the magnitude as the radial distance from the center of the circle chart, and plot the phase angle $\theta$ as the angular displacement from the positive real axis (measured counterclockwise). Using MATLAB, we plot the logarithmic magnitude of Eq. (4.1) evaluated at the symmetry phase and display the real and imaginary parts, as shown in Fig. S6c. Because the system intends to operate in the symmetry phase under normal conditions, features associated with the broken phase are omitted here. At the system’s eigenfrequencies, both the real and imaginary parts of $S_{11}$ become zero. This indicates that $S_{11}$ should coincide with the center of the Smith chart at these frequencies. Consequently, within the plotted frequency range, the $S_{11}$ trajectory passes through the Smith-chart center twice.

Fig. S6 a Photo of the APT-symmetric EP-enhanced PZT sensor. b Photo of measuring the $S_{11}$ curve of this sensor under the symmetry phase. c The curves of $\left| S_{11} \right|$ in decibels, $Im (S_{11})$ and $Re (S_{11}),$ indicating that when the system is in the symmetry phase, the minimum points of the $\left| S_{11} \right|$ and the zeros of both $Im (S_{11})$ and $Re (S_{11})$ are coincident.

Then we use a vector network analyzer (VNA) to simulate it. We find that as *R* decreases, the system’s $S_{11}$resonance peak evolves from a degenerate state [Fig. S7a(i)] to a split state [Figs. S7a(iii), S7a(iv)], indicating that the APT system transitions from the broken phase (degeneracy) to the symmetry phase (splitting). Concurrently examining the Smith chart, we observe that in the broken phase [Fig. S7b(i)] the curve on the chart exhibits no intersection, whereas as *R* is progressively reduced and the system enters the symmetry phase [Figs. S7b(iii), S7b(iv)], the curve looped, and a single intersection node appears. This phenomenon corresponds to the simulation that the $S_{11}$ would pass through the center twice mentioned above, and the Smith chart trace would form a self-intersection precisely when the system is in the symmetry phase. With further decreases in *R*, this loop grows progressively larger [Fig. S7a(iv)], signaling a deepening of the $S_{11}$ resonance splitting [Fig. S7b(iv)]. This indicates that the smaller the self-intersection loop formed by the $S_{11}$ locus on the Smith chart, the closer the system is to the EP.

During the above procedure, we further find that when the system maintains APT symmetry and meets impedance matching, the node always resides at the center of the Smith chart [Fig. S7c(i)] when it is in the symmetry phase [Fig. S7c(ii)]; conversely, when the node deviates from the center [Fig. S7c(iii)], which means the mismatch of the impedance, APT symmetry is thus destructed [Fig. S7c(iv)].

Fig. S7. As the parameter *R* decreases, the curves of $\left| \boldsymbol{S}_{\boldsymbol{11}} \right|$ in decibels (a) and its Smith chart (b) vary from broken phase (i), EP (ii) to symmetry phases [(iii) and (iv)] successively. c The Smith chart (i) and curve of $\left| \boldsymbol{S}_{\boldsymbol{11}} \right|$ (ii) in an impedance-matching system, and the Smith chart (iii), and the curve of $\left| \boldsymbol{S}_{\boldsymbol{11}} \right|$ (iv) in an impedance-mismatching system.

These observations provide direct evidence for Eq. (4.1), which predicts that when the system is in the APT-symmetric phase, both the real and imaginary parts of $S_{11}$ vanish. These observations provide the basis for parameter tuning: A high-precision resistance meter (YHXY-002R, with a resolution of 0.1 mΩ) is interfaced to the measurement pins shown in Fig. S6a to enable real-time tuning of $R_{s3}.$ First, the initial temperature $T_{0}$ is determined based on the target thermometry scenario. Following the exceptional line illustrated in Fig. 2f of the main text, an appropriate system parameter *R* is selected to tune the system into close proximity to the EP near $T_{0}.$ Given that the system’s coupling resistance $R_{3}=R_{s3}-Z_{0}=-R$ and the characteristic impedance $Z_{0}$ is 50 Ω, the required value of $R_{s3}$ is strictly defined by $R_{s3}=Z_{0}-R.$ The employed variable resistors $R_{\text{s1,2,}3}$ feature cross-head slots (Fig. S6a), allowing their resistance to be manually tuned with a screwdriver. Once $R_{s3}$ is fixed, $R_{\text{s1}}$ and $R_{\text{s2}}$ are coarsely adjusted to form a self-intersecting loop on the Smith chart, as depicted in Fig. S7c(iii). Finally, $R_{\text{s1}}$ and $R_{\text{s2}}$ are finely tuned to align this intersection precisely at the center of the Smith chart, as shown in Fig. S7c(i). This procedure successfully meets the demand of $R_{s1}+R_{1}=R_{s2}+R_{2}=R_{s3}-Z_{0}=R$ and configures the system to operate at the EP associated with the initial temperature $T_{0}.$

**Supplementary Note V: Preparations for the LOD and SNR measurement experiment**

As discussed in the main text, we go beyond Allan deviation-based estimates of the limit of detection (LOD) and signal-to-noise ratio (SNR) by conducting temperature-dependent experiments to measure them directly. To this end, we acquire temperature step profiles as shown in Figs. 4c and 4d, using a water-bath heating platform that provides a high degree of stability and precision. The setup employed in this study is illustrated in Fig. S8a. The thermostatic water-bath device used in our experiments maintains a temperature stability of ​​± 0.02 K​ within the range of 288 K to 358 K. The experimental setup consists of one large beaker, one small beaker, a constant-temperature heating stage, and a piece of heat insulating foam. During the experiment, the thermocouple and the test device are placed inside the small beaker. The small beaker is then positioned inside the large beaker, which is filled with an appropriate amount of distilled water. It is critical to ensure that the inner part of the small beaker remains completely dry throughout the experiment to prevent any potential short circuits caused by water ingress. Once all components, including the device, water, and setup, are in place, the opening of the large beaker is tightly sealed with the insulating foam to minimize heat exchange with the external environment. A small hole is made in the foam to allow the wires to pass through. After all preparations are complete, the heating stage is turned on, and the computer is connected to the thermocouple to begin recording temperature data.

The temperature captured by the laptop and the average temperature curves are plotted in Fig. S8b. Here we likewise present only the curves after the temperature has stabilized, omitting the heating ramps. As illustrated in Fig. S8b, within each stable-temperature​ interval, we record the frequency​ data on the computer. Although the temperature inside exhibits noticeable fluctuations, the 0.03 K​ temperature steps remain clearly distinguishable.

Fig. S8. a Setups of the noise robustness experiment. b Acquired temperature in the small beakers (blue) and its average (red).

**Supplementary Note VI: The sensitivity comparison experiments between the EP-enhanced PZT sensor and the single PZT sensor.**

The photos of the sensitivity comparison experiment mentioned in the main text are plotted in Figs. S9a and S9b. In the Fig. S9a, The two PZTs, $X_{1}$ and $X_{2},$ are temporarily fixed in two identical fixtures, and they are connected to the APT PCB through black coaxial lines, which include SMA ports and adjustable resistors $R_{\text{s1}},$ $R_{\text{s2}},$ and $R_{\text{s3}}.$ The VNA is used here for acquiring the $S_{11}$ curves. A similar experiment setup for obtaining the data of a single PZT sensor is depicted in Fig. S9b. We applied a PZT $X_{2}$ connected with a PZT PCB only. The PZT PCB used here includes SMA ports and impedance matching of 50 Ω, and is connected with a VNA to obtain its $S_{11}$ curves. The same computers connected with the same thermocouples in these two pictures are used for acquiring the real-time temperature data.

Fig. S9. a Photo of the experiment setup of the EP-enhanced PZT sensor. b Photo of the experiment setup of the single PZT sensor. We apply only PZT $\boldsymbol{X}_{\boldsymbol{2}}$ here, and it is connected to the PZT PCB, which includes impedance matching of 50 Ω, and the VNA is used for monitoring its $\boldsymbol{S}_{\boldsymbol{11}}$ curve, either.

The detailed specifications of the two PZTs are listed in the table. S1 and table. S2 respectively. The two PZTs are fabricated by JIAKANG ELECTRONICS and are intentionally chosen with distinct material properties to enhance sensing performance.

Table S1. The specification of the PZT element $\boldsymbol{X}_{\boldsymbol{1}}\boldsymbol{.}$

| $X_{1}$ | Materials | Ceramic layer | PZT-4 |
| --- | --- | --- | --- |
|  |  | Electrode layer | Silver |
|  | Apparent density | *ρ* (10^3^ kg/m^3^) | 7.5 |
|  | Relative dielectric constant | *ε*_33_*^T^/ε*_0_ | 1400 |
|  | Coupling Coefficient | *k*_p_ | 0.65 |
|  |  | *k*_33_ | 0.70 |
|  |  | *k*_31_ | 0.38 |
|  | Piezoelectric Constants | *d*_33_ (10^−12^ C/N) | 320 |
|  |  | *d*_31_ (10^−12^ C/N) | −150 |
|  | Mechanical Quality Factor | Q_m_ | 1000 |
|  | Size | Thickness (mm) | 0.13 for the ceramic layer  0.01 for two electrode layers |
|  |  | Dia. (mm) | 10.90 |
|  | BVD model @ 300.9 K | / | *C*_0_: 6.503 nF; *C*_1_: 2.358 nF; *L*_1_: 241.47 μH; *R*_1_: 0.55 Ω |

Table S2. The specification of the PZT element $\boldsymbol{X}_{\boldsymbol{2}}\boldsymbol{.}$

| $X_{2}$ | Materials | Ceramic layer | PZT-5A |
| --- | --- | --- | --- |
|  |  | Electrode layer | Silver |
|  | Apparent density | *ρ* (10^3^ kg/m^3^) | 7.8 |
|  | Relative dielectric constant | *ε*_33_*^T^/ε*_0_ | 1350 |
|  | Coupling Coefficient | *k*_p_ | 0.55 |
|  |  | *k*_33_ | 0.68 |
|  |  | *k*_31_ | 0.32 |
|  | Piezoelectric Constants | *d*_33_ (10^−12^ C/N) | 306 |
|  |  | *d*_31_ (10^−12^ C/N) | −120 |
|  | Mechanical Quality Factor | Q_m_ | 1500 |
|  | Size | Thickness (mm) | 0.13 for the ceramic layer  0.01 for two electrode layers |
|  |  | Dia. (mm) | 10.90 |
|  | BVD model @ 300.9 K | / | *C*_0_: 5.416 nF; *C*_2_: 1.880 nF; *L*_2_: 316.72 μH; *R*_2_: 0.56 Ω |

**Supplementary Note VII: The experiments of robustness of the APT-EP framework against intrinsic fabrication discrepancies in PZTs**

By introducing the temporal CMT theory analysis in Supplementary Note I, we have theoretically proved that the APT structure requires that the two resonant frequencies should have different values to bring the frequency detuning, which provides theoretical support to conveniently choose two PZTs with different resonant frequencies for constructing this EP-enhanced PZT sensor. Here, to explicitly demonstrate the relaxed degeneracy requirements thanks to the APT structure, we choose a batch of 6 PZT elements (denoted as $X_{11}$ to $X_{32})$ and randomly combine these PZTs into three distinct pairs, and using fixtures shown in Fig. S9 and some adjustable resistors to construct the APT-symmetric architectures. As summarized in the Table. S3, these samples exhibit natural variations in their geometric and electrical parameters extracted from the BVD equivalent through Supplementary Note II. For instance, the thickness ranges from 146 μm to 153 μm, and the series resonance frequency $(f_{s})$ fluctuates between 205.75 kHz and 209.82 kHz due to their fabrication discrepancies.

**Table. S3.** The specification and electrical parameters of some PZT elements.

| PZT elements | | | | | | | | | |
| --- | --- | --- | --- | --- | --- | --- | --- | --- | --- |
|  | Material | Thickness (μm) | Dia. (mm) | *f*_s_  (kHz) | *f*_p_  (kHz) | *C*_0_  (nF) | *C*_1_  (nF) | *L*_1_  (μH) | *R*_1_  (Ω) |
| *X*_11_ | PZT-5A | 146 | 10.91 | 209.54 | 244.35 | 6.482 | 2.333 | 247.317 | 0.426 |
| *X*_12_ | PZT-5A | 152 | 10.87 | 205.88 | 240.79 | 5.344 | 1.966 | 303.987 | 0.465 |
| *X*_21_ | PZT-5A | 149 | 10.92 | 209.82 | 244.91 | 6.133 | 2.223 | 258.839 | 0.331 |
| *X*_22_ | PZT-5A | 147 | 10.98 | 208.30 | 241.09 | 7.363 | 2.500 | 233.480 | 0.265 |
| *X*_31_ | PZT-5A | 153 | 10.86 | 205.75 | 240.58 | 5.495 | 2.018 | 296.523 | 0.316 |
| *X*_32_ | PZT-5A | 150 | 10.94 | 206.33 | 241.47 | 5.207 | 1.925 | 309.091 | 0.479 |

Under a constant temperature of 300.9 K, the intrinsic frequency detuning for each pair remains constant. By tuning the external resistors $R_{s1,2,3},$ we dynamically varied the coupling strength $\kappa.$ As shown in the Figs. S10a, S10c, and S10e, the measured eigenfrequencies (circles) of all three randomly paired APT systems closely follow the theoretical calculations (solid lines). Subsequently, we tuned the resistors of the three systems to change their parameter *R* to 6.85 Ω, 2.31 Ω, and 1.11 Ω, respectively, to reach their specific EPs $(\kappa_{\text{EP}}),$ and artificially applied a perturbation $\Delta\kappa=\kappa-\kappa_{\text{EP}}$ respectively. The results of eigenfrequency splitting versus the perturbation $\Delta\kappa$ are plotted in Figs. S10b, S10d, and S10f. Evidently, the logscale insets in these figures demonstrate an excellent fit between the experimental data and theoretical models, robustly maintaining a fractional scaling slope of approximately 0.5 across all three different pairs, demonstrating that these systems preserve the characteristic square-root dependence $\Delta f\propto\Delta\kappa^{1/2}.$

These results demonstrate the robustness of the APT-EP framework against intrinsic fabrication discrepancies, experimentally validating the relaxed requirements for PZT selection.

Fig. S10. a, c, e Measured (circles) and theoretical (solid lines) eigenfrequencies of three randomly combined PZT pairs as a function of the coupling strength $\boldsymbol{\kappa}$ at a constant temperature of 300.9 K. b, d, f The corresponding eigenfrequency splitting versus the applied perturbation $\Delta\boldsymbol{\kappa=\kappa}\boldsymbol{-}\boldsymbol{\kappa}_{\text{EP}}$ when the three systems are tuned to their respective EPs (*R* = 6.29 Ω, 2.31 Ω, and 1.11 Ω). The logscale insets confirm the robust preservation of the slope ≈ 0.5 fractional scaling slope across these mismatched pairs.

Supplementary Note VIII: The sensitivity of EP-enhanced PZT sensor to the drift of resistors

In practical operations, resistance drift is inevitable due to temperature drift, precision limits of the surface-mount adjustable resistors, or manual tuning steps. Consequently, these minute discrepancies induce a slight initial asymmetry within the APT system. Therefore, in the following section, we systematically analyze the stability and robustness of the system against resistance drift under these two specific scenarios.

Under the initial temperature $T_{0},$ the minute drift induced by external resistors $R_{s1,2,3}$ will finally change the equivalent resistances of each branch shown in Fig. S1. We assume that $R_{1,2}=R(1+\eta_{1,2})$ and $R_{3}=-R(1+\eta_{3}),$ with $\left| \eta_{1,2,3} \right|\ll1.$ Note that the original EP rigorously corresponds to a specific initial temperature $T_{0},$ a minuscule drift in the coupling resistance $R_{3}$ directly modifies the coupling coefficient $\kappa_{T_{0}}^{'}=\kappa_{T_{0}}(1+\eta_{3})$ of the system, thereby breaking the original EP condition $(\epsilon_{T_{0}}=\kappa_{T_{0}}\neq\kappa_{T_{0}}^{'}).$ Consequently, the system shifts away from its initial EP state now, and the actual EP corresponds to a new initial temperature ${T_{0}}^{'}.$ From Eq. (2.18), we have

${T_{0}}^{'}=\frac{b_{1}-R(1+\eta_{3})b_{2}}{R(1+\eta_{3})a_{2}-a_{1}}.$

Interestingly, for the EP-enhanced PZT sensor operating at the EP, when $\eta_{3}>0,$ the condition $\epsilon_{T_{0}}<\kappa_{T_{0}}^{'}$ is satisfied, which inevitably drives the system into the broken phase, and consequently causes the frequency splitting and sensitivity to vanish. Fortunately, this condition can be avoided in practice. The initial temperature $T_{0}$ of the EP-enhanced PZT sensor is intentionally set slightly higher than the target temperature $T_{T},$ as illustrated in Fig. S11a. This specific configuration ensures that the system operates in ultra-close proximity to the exact EP while maintaining its operation within the symmetry phase, thereby minimizing the inevitable degradation in its sensitivity. Under such circumstances, even if $\eta_{3}$ inadvertently exhibits a positive value due to manual configuration errors, the system possesses sufficient robustness to tolerate this variation in practical operational scenarios, thereby guaranteeing that it remains stably within the symmetric phase. Furthermore, as detailed in SM note IV, we employ a high-precision resistance meter (YHXY-002R) with a resolution of 0.1 mΩ to accurately calibrate the resistance of $R_{\text{s3}}.$ This approach guarantees the tuning precision of $R_{\text{s3}},$ thereby effectively confining the manual configuration error to a narrow range of ± 0.01 Ω. Consequently, in practical applications, the system consistently operates within the symmetry phase, the effect of sensitivity enhancement produced by APT-EP structure remains effective.

Additionally, due to the asymmetric disturbance induced by resistance $R_{1,2},$ the APT symmetry is also disturbed. Conversely, resistance variations of $R_{1,2}$ in the two resonant branches only affect the total gain and loss balance; they do not induce any changes in the coupling coefficient, nor do they affect the intrinsic frequency detuning $\epsilon_{T_{0}}$ of the system. However, they introduce the imaginary components to the Hamiltonian and the eigenfrequencies. As a result, by applying the CMT, the new Hamiltonian writes

$H_{N}=\left( \begin{matrix} -(\epsilon_{T_{0}}^{'}-\delta^{'}{T_{0}}^{'}a_{1})-i\kappa_{T_{0}}(\eta_{1}-\eta_{3}) & i[\kappa_{T_{0}}^{'}-\delta^{'}{T_{0}}^{'}a_{2}R(1+\eta_{3})] \\ i[\kappa_{T_{0}}^{'}-\delta^{'}{T_{0}}^{'}a_{2}R(1+\eta_{3})] & \epsilon_{T_{0}}^{'}-\delta^{'}{T_{0}}^{'}a_{1}-i\kappa_{T_{0}}(\eta_{2}-\eta_{3}) \end{matrix} \right).$

Here, $\epsilon_{T_{0}}^{'}$ and $\kappa_{T_{0}}^{'}$ represent the frequency detuning and coupling strength within a new initial temperature ${T_{0}}^{'},$ and under the EP condition, $\epsilon_{T_{0}}^{'}=\kappa_{T_{0}}^{'}=\epsilon_{T_{0}}(1+\eta_{3}).$ $\delta^{'}$ is the perturbation, and $\delta^{'}=\left| {T_{0}}^{'}-T_{0}(1-\delta) \right|/{T_{0}}^{'}.$As a result, the eigenfrequency splitting could be expressed as

$\Delta f_{N}\approx2f_{0}\sqrt{\left[ -2{T_{0}}^{'}a_{1}\epsilon_{T_{0}}\left( 1+\eta_{3} \right)+2R{T_{0}}^{'}a_{2}\epsilon_{T_{0}}\left( 1+\eta_{3} \right)^{4} \right]\delta^{'}-8\eta_{3}\epsilon_{T_{0}}^{2}+i\epsilon_{T_{0}}\left( \eta_{1}-\eta_{2} \right)\left[ -{T_{0}}^{'}\delta^{'}a_{1}+\epsilon_{T_{0}}\left( 1+\eta_{3} \right) \right]}.$

As is evident from the expression, the eigenfrequency splitting of the system is dependent on the variable $\eta_{3}$ and the difference term $\eta_{1}-\eta_{2}.$ Figs. S11b and S11c show the $S_{11}$ curves under different values of $\eta_{1}-\eta_{2}$ and $\eta_{3}$ respectively. These curves demonstrate that the eigenfrequencies variation is mainly caused by the change of $\eta_{3},$ while the variation of $\eta_{1}-\eta_{2}$ has almost no difference to the eigenfrequencies, as depicted in Fig. S11d, which demonstrates the frequency splitting versus $\eta_{1}-\eta_{2}$ (red line and dots) and $\eta_{3}$ (blue line and dots) respectively. Interestingly, as $\left| \eta_{1}-\eta_{2} \right|$ increases, the Q factor of both resonant peaks decreases sharply, and one can readily observe that when $\eta_{1}-\eta_{2}>0,$ the Q factor of the left resonant peak experiences a more pronounced degradation compared to the right peak. Conversely, when $\eta_{1}-\eta_{2}<0,$ the right one exhibits a more significant decrease in its Q factor. Consequently, the variation of $\eta_{3}$ plays the dominant role in governing the changes in the eigenfrequency splitting of the system.

Fig. S11. a The initial temperature $\boldsymbol{T}_{\boldsymbol{0}}$ is intentionally selected to be higher than the target temperature $\boldsymbol{T}_{\boldsymbol{T}}\boldsymbol{,}$ for the purpose of operating the system within the symmetry phase. A set of different $\boldsymbol{S}_{\boldsymbol{11}}$ curves of the EP-enhanced PZT sensor under the variation of $\boldsymbol{\eta}_{\boldsymbol{1}}\boldsymbol{-}\boldsymbol{\eta}_{\boldsymbol{2}}$ (b) and $\boldsymbol{\eta}_{\boldsymbol{3}}$ (c) respectively. d The frequency splitting of the systems versus the same variation induced by $\boldsymbol{\eta}_{\boldsymbol{1}}\boldsymbol{-}\boldsymbol{\eta}_{\boldsymbol{2}}$ (red line and dots) and $\boldsymbol{\eta}_{\boldsymbol{3}}$ (blue line and dots) respectively. e TCF of our system (APT-PZT) and a single PZT sensor versus $\boldsymbol{\eta}_{\boldsymbol{3}}\boldsymbol{.}$ $\boldsymbol{\eta}_{\boldsymbol{3}}\boldsymbol{=0}$ represents the original EP, and as $\boldsymbol{\eta}_{\boldsymbol{3}}$ decreases, the TCF of the EP-enhanced PZT sensor decreases sharply.

The sensitivity of the EP-enhanced PZT sensor under drifts of resistors, represented by TCF, is written as follows:

$\text{TC}\text{F}_{N}\approx\frac{-\sqrt{\epsilon_{T_{0}}}\left\{ 2Ra_{2}\left( 1+\eta_{3} \right)^{4}+ia_{1}\left[ \eta_{1}-\eta_{2}+2i\left( 1+\eta_{3} \right) \right] \right\}}{\sqrt{-2\epsilon_{T_{0}}\eta_{3}-i\left( \eta_{1}-\eta_{2} \right)\left[ -{T_{0}}^{'}\delta^{'}a_{1}+\epsilon_{T_{0}}\left( 1+\eta_{3} \right) \right]+2{T_{0}}^{'}\delta^{'}\left( 1+\eta_{3} \right)\left[ -a_{1}+Ra_{2}\left( 1+\eta_{3} \right)^{3} \right]}}.$

As analyzed above, the $\eta_{3}$ is the primary factor responsible for the eigenfrequency splitting. As a result, we set $\eta_{1}-\eta_{2}=0,$ and subsequently rewrite Eq. (8.4) to

$\text{TC}\text{F}_{N}(\eta_{3})\approx\frac{-2\sqrt{\epsilon_{T_{0}}}\left[ Ra_{2}\left( 1+\eta_{3} \right)^{3}-a_{1} \right]\left( 1+\eta_{3} \right)}{\sqrt{-2\epsilon_{T_{0}}\eta_{3}+2{T_{0}}^{'}\delta^{'}\left( 1+\eta_{3} \right)\left[ -a_{1}+Ra_{2}\left( 1+\eta_{3} \right)^{3} \right]}}.$

Assume that the $\delta=0.003,$ representing $\left| \Delta T \right|=1\text{ K}$ at $T_{0}=335.5\text{ K,}$ the evolution of $\left| \text{TC}\text{F}_{N}(\eta_{3}) \right|$ as a function of $\eta_{3}$ is depicted in Fig. S11e; meanwhile, we also represent the TCF of a single PZT sensor versus $\eta_{3}$ here for comparison. Here, the $\left| \text{TC}\text{F}_{N}(\eta_{3}) \right|$ and the TCF of the single PZT sensor has been normalized to $\left| \text{TC}\text{F}_{N}(0) \right|.$

It is evident that the normalized TCF of a single PZT sensor is 0.087, generally lower than the EP-enhanced PZT sensor; when the EP-enhanced PZT sensor works at EP, the value is 1; with the decline of $\eta_{3},$ the $\left| \text{TC}\text{F}_{N}(\eta_{3}) \right|$ decreases rapidly. When $\eta_{3}=0.05,$ the TCF is decreased to 0.32, still significantly better than a single PZT sensor.

However, the resistance tuning of our EP-enhanced PZT temperature sensor employs a methodology that combines a high-precision resistance meter (YHXY-002R, with a resolution of 0.1 mΩ) with Smith chart monitoring (detailed in Supplementary Note IV). This largely guarantees the precision of the parameter *R* tuning. Furthermore, the adjustable resistors utilized in our circuit are the NIDEC ST-32EA, which features a temperature coefficient of resistance (TCR) of ± 250 ppm/K. This indicates that even over a substantial temperature difference of 100 K, the resistance fluctuation is merely 2.5%. Moreover, our device operates exclusively within a very narrow temperature range (approximately ± 10 K) around the initial temperature $T_{0}.$ Consequently, the influence on resistance caused by temperature variations is negligible. Therefore, our EP-enhanced PZT temperature sensor possesses high robustness.

**Supplementary References**

1. Kononchuk, R., Cai, J., Ellis, F., Thevamaran, R. & Kottos, T. Exceptional-point-based accelerometers with enhanced signal-to-noise ratio. *Nature* **607**, 697–702 (2022).

2. Haus, H. A. & Huang, W. Coupled-mode theory. *Proc. IEEE* **79**, 1505-1518 (1991).

3. Chen, Y. *et al.* A systematic analysis of the radial resonance frequency spectra of the PZT-based (zr/ti = 52/48) piezoceramic thin disks. *J. Adv. Ceram.* **9**, 380–392 (2020).

4. IEEE standard definitions and methods of measurement for piezoelectric vibrators. *IEEE Std. No.177*, 1 (1966).

5. IEEE standard on piezoelectricity. *ANSI/IEEE Std. 176-1978*, 1-58 (1978).
